# Transforming acidic coiled-coil-containing protein 3-mediated lipid metabolism reprogramming impairs CD8^+^ T-cell cytotoxicity in hepatocellular carcinoma

**DOI:** 10.1038/s41392-025-02367-9

**Published:** 2025-08-28

**Authors:** Ying Li, Zule Chen, Dongdong Wang, Wei Du, Ningqi Zhu, Xiaotian Shen, Xiang Mao, Yinghan Su, Lunxiu Qin, Diyu Chen, Huliang Jia

**Affiliations:** 1 Department of General Surgery, Huashan Hospital (Baoshan Branch), Fudan University, Shanghai, China; 2 Hepatobiliary Surgery Center, Department of General Surgery, Huashan Hospital, Fudan University, Shanghai, China; 3 Cancer Metastasis Institute, Fudan University, Shanghai, China; 4 Department of Radiology, Huashan Hospital (Baoshan Branch), Fudan University, Shanghai, China; 5 Department of Breast Surgery, Obstetrics and Gynecology Hospital of Fudan University, Shanghai, China

**Keywords:** Cancer metabolism, Cancer microenvironment, Drug development, Tumour immunology, Tumour biomarkers

## Abstract

Recent evidence has highlighted immune checkpoint inhibitors as among the most promising immunotherapies for various malignancies. However, a significant proportion of HCC patients exhibit poor responses. Lipid metabolic heterogeneity is considered a key driver of cancer progression. However, the role of lipid metabolic reprogramming in HCC immunotherapy resistance remains poorly understood. Herein, we aimed to illuminate the potential relationship between lipid metabolic reprogramming and ICI resistance and provide novel strategies to increase the HCC immunotherapy response. Patients who received PD-1/PD-L1 inhibitors were enrolled. The effect of TACC3 on the tumor microenvironment was validated via single-cell RNA sequencing in HCC-bearing mouse models. Targeted metabolomics was performed to analyze the regulatory role of TACC3 in HCC metabolism. To address HCC immunotherapy resistance, we developed a targeted nucleic acid therapeutic utilizing N-acetylgalactosamine (GalNAc) to conjugate siTACC3. Through clinical cohort analysis, we found that TACC3 was overexpressed in HCC patients with poor response to immunotherapy. Furthermore, we demonstrated that silencing tumor-derived TACC3 optimizes the cytotoxicity of infiltrating CD8^+^ T lymphocytes. Both in vitro and in vivo assays suggested that TACC3 maintains ACSL4-mediated polyunsaturated fatty acid (PUFA) metabolism in HCC cells. Additionally, TACC3 accelerates ACSL4 expression by interacting with LARP1 and PABPC1, which stabilize ACSL4 mRNA. The results of preclinical models demonstrated the satisfactory efficacy of GalNAc-conjugated siTACC3 combined with PD-1 inhibitor therapy for HCC. In summary, tumor-derived TACC3 impairs the tumor-killing activity of CD8^+^ T lymphocytes through PUFA metabolism-associated crosstalk. Targeting TACC3 represents a novel and practicable strategy to augment ICI efficacy against HCC.

## Introduction

Recently, immunotherapies, primarily represented by immune checkpoint inhibitors (ICIs), have led to significant advancements in the systemic treatment of hepatocellular carcinoma (HCC).^1^ However, treatment resistance has severely decreased the clinical efficacy of ICIs.^2^ As shown by the follow-up data of the CheckMate 459 clinical trials, compared with that of subjects in the sorafenib treatment group, the objective response rate (ORR) of subjects in the nivolumab treatment group increased by only 8% to 15%, and the median overall survival (mOS) was extended by only 2.3 months.^3^ Thus, identifying new targets to increase immunotherapy sensitivity has emerged as an imminent challenge.

Competent immune cells are a prerequisite for achieving optimal therapeutic outcomes with ICIs. However, cytotoxic T lymphocytes (CD8^+^ T cells), the primary immune cell subset responsible for tumor clearance, often exhibit a dysfunctional phenotype characterized by decreased production of tumor-killing factors such as interferon-γ (IFN-γ), impaired proliferative potential, and elevated expression of exhaustion markers such as cytotoxic T lymphocyte-associated antigen 4 (CTLA-4).^4^ In light of this, the primary mechanism of current ICIs involves binding to immune checkpoints and blocking downstream signaling pathways, thereby reinvigorating CD8^+^ T cells. Nevertheless, monotherapy with ICIs frequently faces challenges such as treatment resistance, unclear responsive populations, and the inability to fully revitalize the cytotoxicity of CD8^+^ T lymphocytes, making it particularly urgent to identify new targets to assist in reactivating CD8^+^ T cells for combination therapy with ICIs.

Interactions between lipid metabolism in cancer cells and CD8^+^ T cells function as key drivers of the impaired tumor clearance ability of this immune subset.^5,6^ Tumor cell ZDHHC palmitoyltransferase 3 (ZDHHC3) stabilizes SREBP cleavage-activating protein (SCAP) via S-acylation at Cys264, thereby inhibiting its ubiquitination. Stabilized SCAP activates sterol regulatory element binding transcription factor 2 (SREBP2), driving cholesterol synthesis gene expression and increasing cholesterol in the tumor microenvironment (TME).^6^ Critically, this excess cholesterol acts as an immunosuppressive factor, blunting CD8^+^ T-cell cytotoxicity by diminishing the release of the effector molecules IFN-γ and granzyme B (GZMB).^5^ The metabolism of PUFAs, critical components of lipid metabolism, is essential for processes such as cell membrane renewal, inflammatory signaling, and nervous system development.^7^ Notably, linoleic acid (LA), a member of the n-6 PUFA family, has the capacity to increase the mitochondrial function of cytotoxic T lymphocytes and reverse exhaustion.^8^ Additionally, systemic PUFA status directly shapes immune cell functionality. Previous clinical evidence has demonstrated that one month of supplementation with high-dose n-3 PUFAs (3.5 g eicosapentaenoic acid [EPA] and 1.75 g docosahexaenoic acid [DHA]) ameliorated mitochondrial bioenergetic dysfunction in the peripheral blood mononuclear cells (PBMCs) of obese individuals, specifically by reducing pathological nonmitochondrial respiration while increasing spare respiratory capacity and the bioenergetic health index. This intervention concurrently normalized elevated proinflammatory (T helper 1, T helper 17, classically activated monocytes) and anti-inflammatory/regulatory (T helper 2, CD4^+^/CD8^+^ regulatory T cells, regulatory B cells) immune subsets, underscoring the capacity of PUFAs to reprogram immunometabolic fitness.^9^ However, the role of tumor-derived PUFAs in impairing CD8^+^ T-cell-mediated tumor surveillance and driving immunotherapy resistance remains poorly understood, particularly their mechanistic impact on T-cell mitochondrial bioenergetics and exhaustion phenotypes, warranting targeted investigation. Tumor cells employ intrinsic regulatory mechanisms to rewire metabolism and establish a suppressive TME, thereby evading T-cell surveillance. For example, tumor cells expressing nucleus accumbens-associated protein-1 (NAC1) upregulate lactate dehydrogenase A (LDHA), leading to increased lactic acid production and the formation of an immunosuppressive TME that impairs T-cell function.^10,11^ Transforming acidic coiled-coil-containing protein 3 (TACC3), an intracellular regulator, mainly modulates mitosis, chromosome stability, and tumor progression.^12,13^ Overexpressed in tumors, TACC3 promotes chemoresistance in pancreatic ductal adenocarcinoma through interaction with kinesin family member 11 (KIF11).^14^ Additionally, TACC3 has been shown to modulate immune responses, as its ablation restores trastuzumab sensitivity in breast cancer (BRCA) by recruiting dendritic and cytotoxic T cells.^15^ Despite these findings, the roles of TACC3 in tumor regulation, immunomodulation, and metabolic reprogramming in liver cancer remain unexplored, as does its potential to overcome resistance to immunotherapy in HCC.

Herein, we demonstrate that TACC3, which is highly expressed in immunotherapy-resistant HCC tissues, reprograms acyl-CoA synthetase long-chain family member 4 (ACSL4)-mediated PUFA metabolism via la-related protein 1 (LARP1) and poly(A)-binding protein cytoplasmic 1 (PABPC1)-regulated ACSL4 mRNA stability. This metabolic reprogramming deprives CD8^+^ T cells of essential PUFAs such as DHA, impairing their antitumor activity. Our findings provide novel insights into how tumor cells evade immune clearance through lipid metabolic rewiring and suggest that TACC3 is a promising target for enhancing immunotherapy efficacy in HCC.

## Results

### TACC3 is overexpressed in nonresponders to HCC immunotherapy

**Fig. 1:**
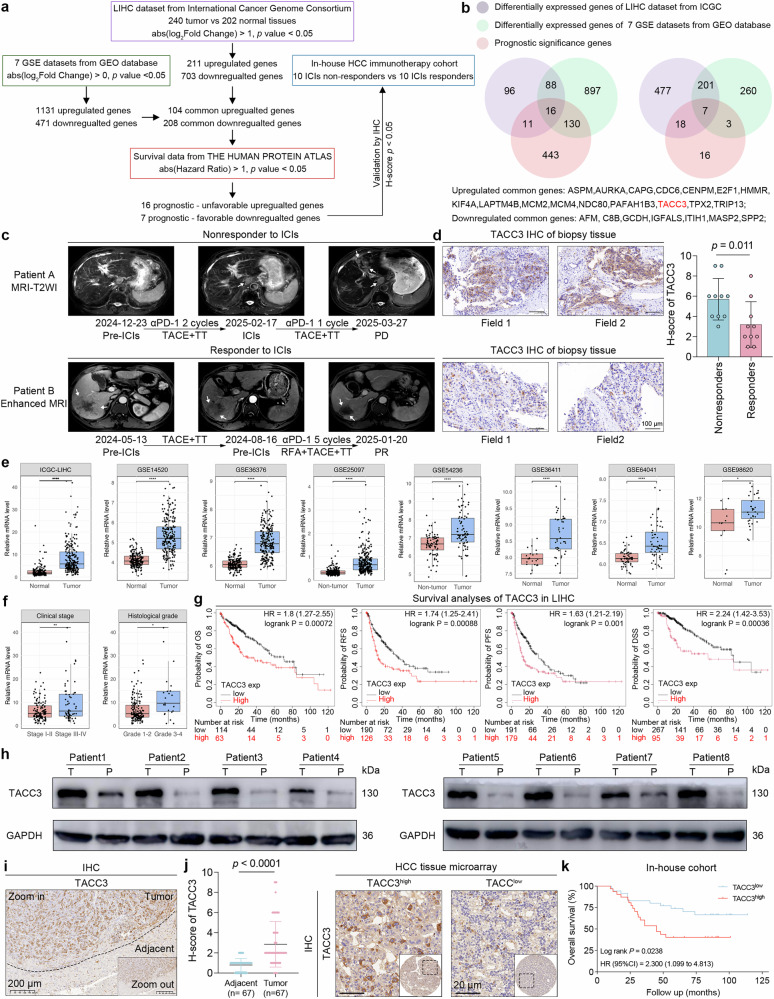
TACC3 is overexpressed in nonresponders to HCC immunotherapy. **a**, **b** Schematic diagram (**a**) and Venn diagram (**b**) of the screening process for the target gene TACC3 in the ICGC-LIHC dataset, 7 GEO datasets, the Human Protein Atlas database, and our in-house immunotherapy cohort. DEGs were detected via the DESeq2 and limma packages in R software. Abs absolute value, HR hazard ratio, ICIs immune checkpoint inhibitors. Details of the H score are provided in the Supplementary Materials. **c**, **d** Representative imaging tests (**c**) and TACC3 IHC evaluation (**d**) of responders and nonresponders to ICIs. Pre before ICI therapy, TACE transarterial chemoembolization, TT targeted therapy, RFA radiofrequency ablation, PD progressive disease, PR partial response. Scale bars for IHC analysis, 100 μm. **e** The relative expression levels of TACC3 in HCC and adjacent nontumor tissues from the ICGC-LIHC cohort and 7 GEO datasets are shown in box plots. **f** The relationship between TACC3 expression and clinical parameters such as clinical stage and histopathological grading was analyzed using the ICGC-LIHC dataset. **g** OS, RFS, PFS, and DSS curves for HCC patients were generated via KMplotter. The optimal cutoff value for TACC3 expression (high vs low) was calculated automatically via the KMplotter platform via the maximally selected rank statistics algorithm. P values were calculated via the log-rank (Mantel‒Cox) test. **h** TACC3 protein expression levels in 8 paired HCC and paratumor tissues were quantified via western blotting analysis. GAPDH was used as a loading control. T tumor tissue, P paratumor tissue. **i** IHC analysis of tissue sections revealed the localization of TACC3 in the HCC tumor tissue and adjacent tissue. Scale bars, 200 μm. **j** TACC3 expression levels in our in-house cohort, which included 67 paired HCC tumors and adjacent normal tissues, were quantified through IHC analysis and H score. Scale bars, 20 μm. **k** Sixty-seven HCC tumor tissues were divided into TACC3-high and TACC3-low-expression groups according to the median TACC3 H score from the IHC analysis. The survival status of these patients was regularly followed up, and survival curves were drawn. The median value of TACC3 IHC staining intensity was set as a cutoff. The P value was calculated as described in (**g**). Data and error bars are presented as the means ± SDs. The data were analyzed via the Wilcoxon signed-rank test (**d**), Student’s t test (**e**, **f**, **j**), and log-rank (Mantel‒Cox) test (**g**, **k**)

To identify novel genes driving resistance to immunotherapies and progression in HCC, we performed systematic profiling for novel drivers among differentially expressed genes (DEGs) via data from the International Cancer Genome Consortium-Liver Hepatocellular Carcinoma (ICGC-LIHC) cohort and seven GEO datasets (GSE14520, GSE36376, GSE25097, GSE54236, GSE36411, GSE64041, and GSE98620), as outlined in the flowchart (Fig. 1a). These DEGs were further filtered via a prognostic gene set for liver cancer downloaded from the Human Protein Atlas (HPA) database (Supplementary Table 1). Through this rigorous process, we identified seven consistently downregulated genes and sixteen shared upregulated genes. Among these DEGs, TACC3 was identified as an understudied factor within the framework of immunotherapy resistance and HCC progression (Fig. 1b). Interestingly, a previous study reported that targeting TACC3 could stimulate immunogenic cell death and increase trastuzumab emtansine responsiveness in BRCA, suggesting a potential role for TACC3 in immune regulation and immunotherapy resistance in HCC.^15^

Through comprehensive imaging evaluation (Fig. 1c) and immunohistochemistry (IHC) validation (Fig. 1d) in our cohort of 20 HCC patients receiving ICI therapy (10 responders vs 10 nonresponders, Supplementary Fig. 1a, b), we identified marked upregulation of TACC3 protein expression in ultrasound-guided biopsy samples from immunotherapy-resistant patients. Stratification by the H score of TACC3 revealed distinct clinical trajectories: the high-TACC3 cohort (n = 10) demonstrated significantly higher disease progression rates (8/10 patients) than did the low-TACC3 cohort (2/10 progression events, Supplementary Fig. 1c). Longitudinal survival data further distinguished these cohorts, with the high-TACC3 subgroup exhibiting both disease progression and mortality in 40% of patients (4/10) and sustained progression-free survival in 80% (8/10) of low-TACC3 patients (Supplementary Fig. 1d). These findings position TACC3 as a potential biomarker and mechanistic contributor to immunotherapy resistance and progression in HCC.

Further analysis of multiple independent HCC datasets revealed that TACC3 mRNA was markedly upregulated in cancer tissues compared with peritumoral samples (Fig. 1e). Additionally, elevated TACC3 expression was significantly correlated with advanced clinical stage and poorer histological grade in the ICGC-LIHC cohort (Fig. 1f). Correlation analysis also demonstrated a robust positive relationship between TACC3 and Ki67 mRNA levels (Supplementary Fig. 1e), corroborating prior evidence of its protumorigenic functions.^13,14,16,17^

Survival analysis suggested that patients with higher TACC3 mRNA levels had worse overall survival (OS), relapse-free survival (RFS), progression-free survival (PFS), and disease-specific survival (DSS) (Fig. 1g). Furthermore, pancancer survival analysis suggested that elevated TACC3 expression predicted poorer OS and RFS in multiple cancers, including pancreatic ductal adenocarcinoma and lung adenocarcinoma (Supplementary Fig. 1f, g).

To verify these expression profile data via bioinformatics analysis, western blotting analysis of eight paired tumor and adjacent nontumor samples revealed that TACC3 was overexpressed in HCC tissues compared with adjacent normal tissues (Fig. 1h and Supplementary Fig. 1h). Consistently, initial IHC analysis of conventional sections revealed significantly greater TACC3 expression in tumor tissues than in adjacent nontumor tissues (Fig. 1i). This result was further validated in an expanded cohort via a tissue microarray containing 67 matched HCC tumor-paracancerous pairs (Fig. 1j). Next, patients were stratified into two groups according to the median TACC3 IHC H score. The relationship between high TACC3 levels and decreased OS was further confirmed through follow-up and survival analysis (Fig. 1k).

Taken together, our results reveal that TACC3 may function as a fundamental factor in HCC resistance to immunotherapy.

### scRNA-seq illuminates the potential immunosuppressive role of TACC3

The findings from our initial analysis prompted us to further investigate the specific immune regulatory effects of TACC3. Bioinformatics analysis of the ICGC-LIHC dataset demonstrated that TACC3 expression was significantly positively correlated with multiple immune checkpoints, including PD-1 and CTLA-4 (Supplementary Fig. 2a). In addition, bioinformatics assessment revealed that TACC3 expression is significantly positively associated with the enrichment of suppressive immune cells, including myeloid-derived suppressor cells (MDSCs), while it is inversely related to the infiltration of cytotoxic immune cells, including natural killer (NK) cells (Supplementary Fig. 2b). Importantly, single sample gene set enrichment analysis (ssGSEA) of the ICGC-LIHC dataset revealed that high TACC3 expression was positively related to the infiltration levels of MDSCs and regulatory T lymphocytes (Supplementary Fig. 2c). Collectively, these data reveal the potential immunosuppressive role of TACC3.

**Fig. 2:**
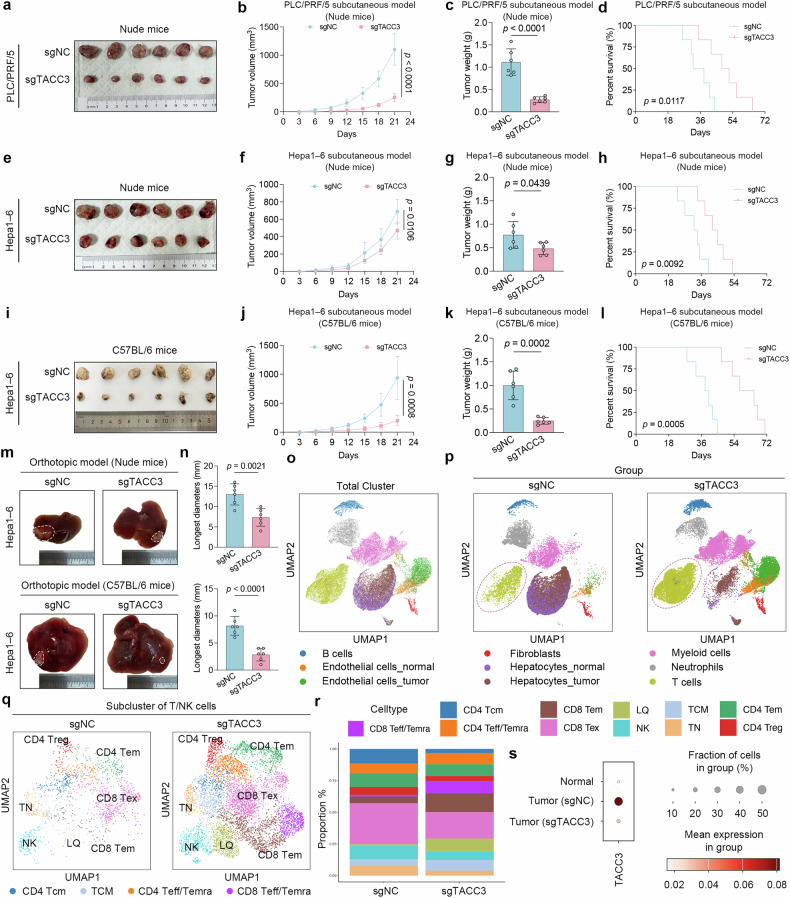
scRNA-seq illuminates the potential immunosuppressive role of TACC3. **a**‒**d** Representative images (**a**), growth curves (**b**), and weights (**c**) of a subcutaneous tumor model established with the human HCC cell line PLC/PRF/5 and nude mice. Parallel experiments were performed, and survival data were collected to obtain survival curves (**d**) (n = 6 per group). **e**‒**h** Representative images (**e**), growth curves (**f**), and weights (**g**) of a subcutaneous tumor model generated from the mouse HCC cell line Hepa1‒6 and nude mice. The survival curve (**h**) was drawn as described previously (n = 6 per group). **i**‒**l** Representative images (**i**), growth curves (**j**), and weights (**k**) of a subcutaneous tumor model generated from Hepa1‒6 cells and C57BL/6 mice. The survival curve (**l**) was drawn as described previously (n = 6 per group). **m**, **n** Representative images of the orthotopic tumor model (**m**) and the longest diameters (**n**) of the orthotopic tumors (n = 6 per group). **o** Overall uniform manifold approximation and projection (UMAP) visualization of scRNA-seq data from orthotopic tumor tissues, color-coded by annotated cell types. **p** UMAP plots showing different cell types in orthotopic tumor tissues of sgNC and sgTACC3 groups. **q** UMAP plot was created by subclustering the T/NK cells of the aforementioned scRNA-seq. CD8 Teff/Temra effector CD8^+^ T cells, CD4 Teff/Temra effector CD4^+^ T cells, Tcm central memory T cells, CD4 Tem effector memory CD4^+^ T cells, CD4 Treg CD4^+^ regulatory T cells, CD8 Tex exhausted CD8^+^ T cells, NK natural killer T cells, CD8 Tem effector memory CD8^+^ T cells, TN naive T cells, LQ low-quality cells. **r** Stacked column chart of the proportions of different T/NK cell subpopulations in the sgNC and sgTACC3 groups. **s** Bubble plot showing the relative TACC3 expression levels in nontumor, sgNC, and sgTACC3 tumor cells. Normal, nontumor cells. Data and error bars are presented as the means ± SDs. The data were analyzed via Student’s t test (**b**, **c**, **f**, **g**, **j**, **k**, **n**) and the log-rank (Mantel‒Cox) test (**d**, **h**, **l**)

To identify whether TACC3 is an immunosuppressive factor in vivo, tumor models were constructed using PLC/PRF/5 and Hepa1‒6 cell lines in which TACC3 was knocked out via CRISPR/Cas9 technology, as validated by western blotting analysis (Supplementary Fig. 3a, b). Subcutaneous tumorigenesis models were constructed via the use of sgNC and sgTACC3 PLC/PRF/5 cells in nude mice. The results indicated that deletion of TACC3 impeded tumor development and extended the OS of the model mice (Fig. 2a‒d), which is in line with previous reports that TACC3 promotes cell proliferation.^14,18^

To further investigate the immune-mediated effects, tumor models were constructed via the subcutaneous injection of sgNC and sgTACC3 Hepa1‒6 cells in both nude and C57BL/6 mice. Tumor growth was more strikingly inhibited in C57BL/6 mice following TACC3 deletion than in nude mice (Fig. 2e‒l). These data illustrate that the more pronounced inhibition of cancer growth in C57BL/6 mice may be attributed to the alleviation of the immunosuppressive function of TACC3 caused by sgTACC3 transfection. In addition, after intratumoral injection of siTACC3, we also observed consistent results in both nude mice and C57BL/6 subcutaneous tumor models (Supplementary Fig. 3c‒e). Q. Zhu et al. similarly demonstrated varying degrees of tumor growth suppression in nude mice compared with their C57BL/6 counterparts, with differential statistical significance attributable to the dual modulatory effects of O-GlcNAcylation on proliferative and immune pathways.^19^ To better mimic the tumor immune microenvironment (TIME) during HCC progression, orthotopic tumor models were further constructed in nude and C57BL/6 mice after establishing subcutaneous models (Fig. 2m). The longer diameter of the orthotopic tumors also demonstrated that ablation of TACC3 more significantly impeded tumor growth in C57BL/6 mice than in nude mice (Fig. 2n).

Recently, single-cell RNA sequencing (scRNA-seq) has provided a more precise understanding of the TIME by enabling the characterization of individual immune cell types and their interactions with other cell types. Thus, to gain deeper insight into immune cell profiling, scRNA-seq was performed on orthotopic liver tumors established using TACC3-knockout or control Hepa1‒6 cells, from two mice per group. As shown in the uniform manifold approximation and projection (UMAP) plot in Fig. 2o, nine main cell types were annotated via canonical markers following quality control and dimensionality reduction clustering (Supplementary Fig. 3f). The scRNA-seq data revealed that the infiltration levels of B and T/NK cells were markedly greater in the sgTACC3 group than in the sgNC group, whereas the trend for neutrophils was the opposite (Fig. 2p). Subclustering the T/NK cells further confirmed that the elimination of TACC3 in tumors increased the proportions of effector CD8^+^ T cells, effector memory CD8^+^ T cells, and central memory CD8^+^ T cells while reducing the ratios of exhausted CD8^+^ T cells and CD4^+^ regulatory T cells (Fig. 2q, r). These results suggest that targeting TACC3 could redirect the suppressive TIME toward an immunotherapy-sensitive TIME with inflammatory characteristics. Additionally, bubble plots of the scRNA-seq data indicated that TACC3 expression is specifically enriched in cancer cells compared with other cell types (Fig. 2s), suggesting that the biological functions of TACC3 in other cell types are not dominant.

In summary, our in vivo experiments and scRNA-seq data demonstrated that TACC3 possesses immunosuppressive functions, which could be achieved through crosstalk with CD8^+^ T cells and negatively impact the TIME.

### TACC3 promotes HCC progression by inhibiting the antitumor immunity of CD8^+^ T cells

To elucidate the specific mechanism by which TACC3 induces a desert-like TIME, we focused on cytotoxic T lymphocytes, which constitute the most enriched immune cell subsets within the TIME and are critical for antitumor immunity. High-level enrichment of CD8^+^ T lymphocytes within tumor tissues predicts a better patient prognosis.^20^ However, cancer cells induce the dysfunction of CD8^+^ T lymphocytes through various mechanisms, thereby creating a suppressive TIME.^21^ For example, a previous study reported that hexokinase domain component 1 (HKDC1) weakens the tumor-clearance activity of cytotoxic T lymphocytes through activating the STAT1/PD-L1 axis in tumor cells.^22^ On the basis of our scRNA-seq findings, we hypothesized that TACC3 shapes a negative TIME in a manner dependent on CD8^+^ T cells.

**Fig. 3:**
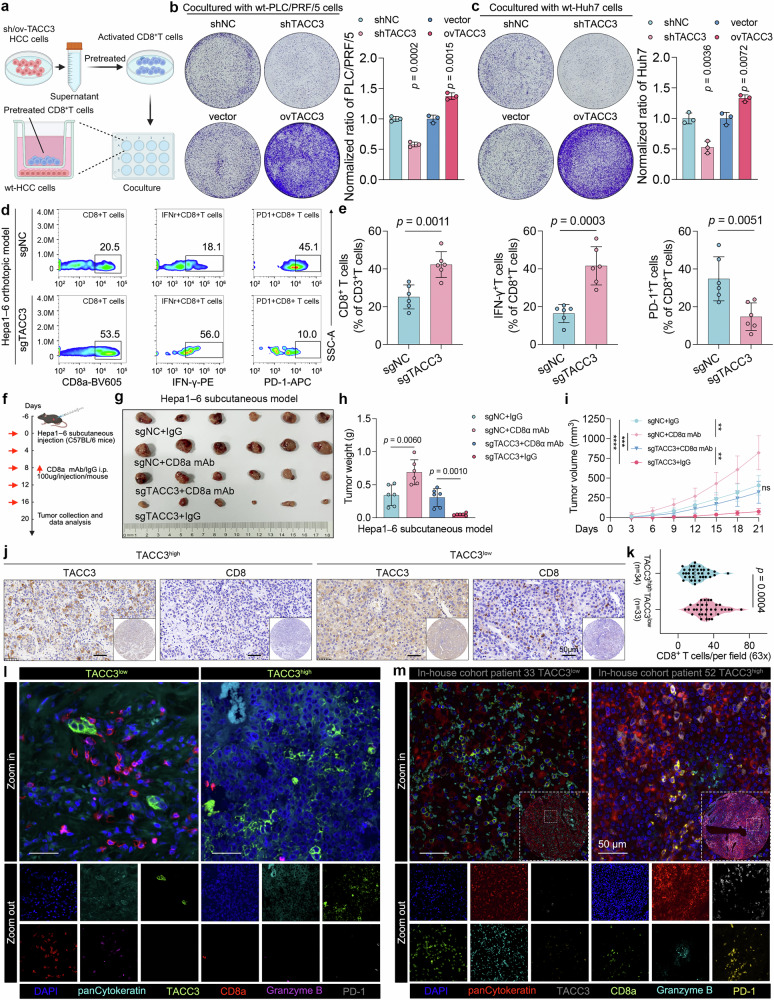
TACC3 promotes HCC progression by inhibiting the antitumor immunity of CD8^+^ T cells. **a**‒**c** Schematic diagrams of the CD8^+^ T-cell-mediated tumor killing assay (**a**), which was conducted by coculturing activated CD8^+^ T cells and wt HCC cells. Before cocultivation, activated CD8^+^ T cells were pretreated with supernatants from PLC/PRF/5 (**b**) or Huh7 cells (**c**) transfected with shTACC3 or ovTACC3. **d**, **e** Flow cytometry analysis was performed on CD45^+^CD3^+^ cells from an orthotopic tumor model (**d**) to detect the infiltration of total CD8^+^ T cells, IFN-γ^+^CD8^+^ T cells, and PD-1^+^CD8^+^ T cells (**e**) in sgNC or sgTACC3 HCC orthotopic tumor tissues (n = 6 per group). **f**, **g** Schematic diagram (**f**) and representative image (**g**) of the subcutaneous tumor model with anti-CD8a mAb administration. The anti-CD8a mAb was intraperitoneally injected every 4 days starting 6 days after the subcutaneous injection of the mouse HCC cell line Hepa1-6 (n = 6 per group). **h**, **i** Weights (**h**) and growth curves (**i**) of subcutaneous tumors as described in (**f**, **g**). **j** CD8a IHC analysis of our in-house cohort (n = 67); patients were divided into TACC3-high and TACC3-low groups according to the median TACC3 H score. Detailed information on the H scores is provided in the Supplementary Materials. **k** Violin plot of CD8a IHC analysis as described in (**j**). Scale bars, 50 μm. **l**, **m** mIHC analysis of TACC3, pancytokeratin, CD8a, GZMB, and PD-1 in an initial cohort of 6 HCC samples (**l**) and the expanded HCC tissue microarray cohort (n = 67) (**m**). For both analyses, patients were stratified into TACC3-high and TACC3-low groups on the basis of the median TACC3 mean fluorescence intensity (MFI) within the respective cohort. Scale bars, 50 μm. Data and error bars are presented as the means ± SDs. The data were analyzed by Student’s t test (**b**, **c**, **e**, **h**, **i**, **k**)

As expected, scRNA-seq feature plots revealed that transfection with sgTACC3 decreased the proportions of PDCD1^+^CD8^+^ T cells, TOX^+^CD8^+^ T cells, and HAVCR2^+^CD8^+^ T cells, which are considered exhausted CD8^+^ T cells that have lost their antitumor activity (Supplementary Fig. 4a).^23^ To evaluate the impact of tumorous TACC3 on CD8^+^ T cells, a CD8^+^ T-cell-mediated tumor killing assay using TACC3-knockdown and TACC3-overexpressing HCC cell lines was subsequently performed (Fig. 3a and Supplementary Fig. 4b‒i). The results revealed that CD8^+^ T cells pretreated with the culture medium (CM) from siTACC3 or shTACC3 HCC cells presented enhanced antitumor function, whereas those pretreated with the CM from ovTACC3 HCC cells presented significantly restricted tumor-killing capacity (Fig. 3b, c and Supplementary Fig. 4j‒l). These findings indicate that TACC3 in HCC cells indirectly impairs the tumor-elimination activity of CD8^+^ T cells in vitro.

Next, flow cytometry analysis of tumor tissues from an in situ HCC model revealed that interference with TACC3 expression in Hepa1‒6 cells increased the ratio of total tumor-infiltrating CD8^+^ T cells and IFN-γ^+^CD8^+^ T cells while clearly decreasing the proportion of PD-1^+^CD8^+^ T cells (Fig. 3d, e and Supplementary Fig. 10a). Furthermore, in subcutaneous tumor models, IHC analysis demonstrated concordant findings with the aforementioned observations irrespective of whether the intervention was administered via intratumoral siTACC3 injection or through subcutaneous implantation of TACC3-knockout cells (Supplementary Fig. 4m‒p). These results revealed that TACC3 creates a suppressive TIME by obstructing the antitumor immunity of CD8^+^ T cells. To further confirm this, rescue experiments were performed utilizing a CD8a monoclonal antibody (mAb) (Fig. 3f), the results of which proved that neutralization of CD8^+^ T cells significantly attenuated the inhibitory effect of sgTACC3 on the development of cancer cells (Fig. 3g‒i), further verifying that TACC3 promotes HCC deterioration partially in a CD8^+^ T-cell-dependent manner.

Furthermore, IHC and multiplex IHC (mIHC) assays were carried out to further confirm the association of TACC3 expression with the antitumor phenotype of cytotoxic T lymphocytes in our clinical cohort. The results revealed that elevated TACC3 expression was inversely correlated with cytotoxic CD8^+^ T lymphocyte infiltration within the TIME (Fig. 3j, k). Moreover, mIHC analysis of six HCC patient samples revealed that the levels of CD8^+^ T-cell infiltration and GZMB secretion were strikingly greater in TACC3-low tumor samples than in TACC3-high tumor samples (Fig. 3l). Consistently, the ratio of PD-1^+^CD8^+^ T lymphocytes was lower in TACC3-low tumor tissues than in control tissues (Fig. 3l). Additional confirmation emerged through the mlHC examination conducted on our tissue microarray, which included an expanded sample of 67 HCC tumor tissues (Fig. 3m).

In summary, we elucidated that TACC3 shapes an immune desert via CD8^+^ T-cell-dependent pathways, thereby facilitating HCC progression.

### TACC3 reprograms PUFA metabolism to impair CD8^+^ T-cell antitumor immunity

Emerging evidence has demonstrated that metabolic crosstalk initiated by tumor cells has crucial effects on the functional dynamics of CD8^+^ T-cell-mediated antitumor ability.^24^ Recent studies have elucidated connections between T-cell dysfunction and tumor cells through discrete facets, such as amino acid communication and glucose metabolic reprogramming.^25,26^ However, the influence of intratumor regulatory element-mediated lipid metabolic plasticity, particularly PUFA homeostasis, on CD8^+^ T-cell effector functions requires comprehensive elucidation.

To comprehensively characterize the metabolic regulatory role of TACC3, we conducted RNA sequencing (RNA-seq) in TACC3-knockdown PLC/PRF/5 cells. Integrated enrichment analyses based on the Kyoto Encyclopedia of Genes and Genomes (KEGG) and gene set enrichment analysis (GSEA) revealed coordinated dysregulation of metabolic processes coupled with immune-related pathway alterations (Supplementary Fig. 5a‒d). These computational results prompted us to conduct a more precise metabolomics analysis to clarify the metabolic regulatory function of TACC3.

**Fig. 4:**
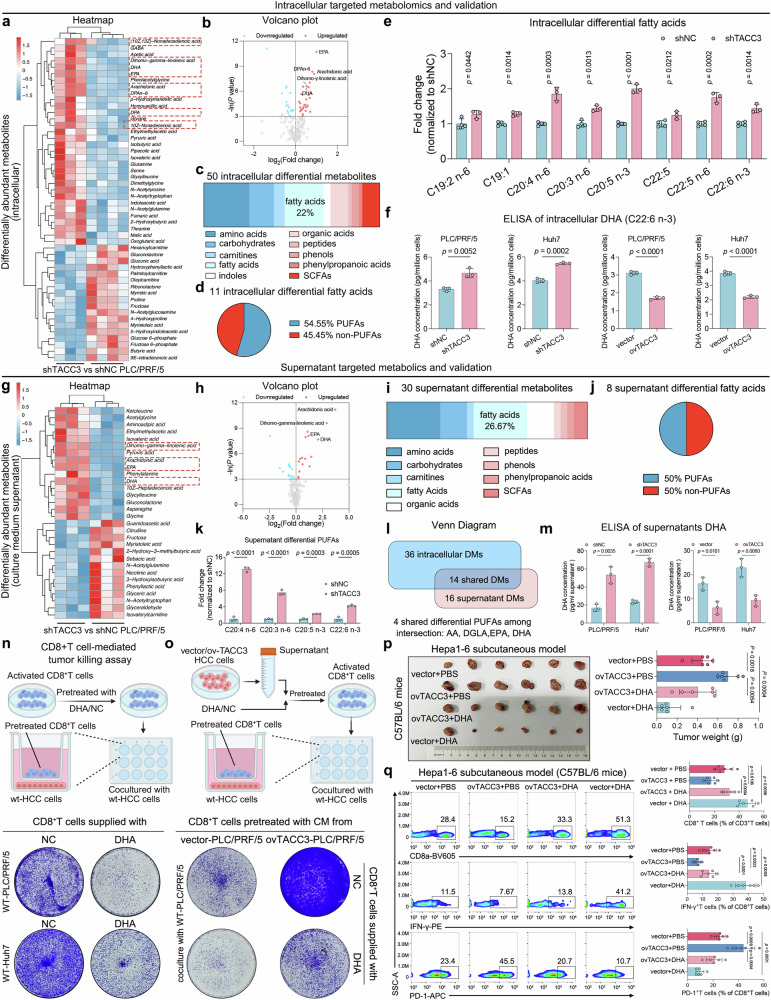
TACC3 reprograms PUFA metabolism to impair CD8^+^ T-cell antitumor immunity. **a**, **b** Heatmap (**a**) and volcano plot (**b**) of targeted metabolomics results showing the intracellular differentially abundant metabolites of PLC/PRF/5 cells (four shNC vs three shTACC3 PLC/PRF/5 cells as one sample in the shTACC3 group was excluded for quality control failure). **c** Bar chart showing the percentages of different types of differential intracellular metabolites. **d** Pie chart showing the percentages of intracellular differential PUFAs and non-PUFAs. PUFAs polyunsaturated fatty acids. **e** Fold-change of intracellular differential fatty acids via our intracellular targeted metabolomics data. **f** Intracellular DHA concentrations in PLC/PRF/5 and Huh7 cells detected via ELISA. **g**, **h** Heatmap (**g**) and volcano plot (**h**) of targeted metabolomics data showing the differentially abundant metabolites in CM supernatants from PLC/PRF/5 cells (shNC, n = 3; shTACC3, n = 3). **i** Bar chart showing the percentages of different types of differentially abundant metabolites in the supernatants. **j** Pie chart showing the percentages of differential PUFAs and non-PUFAs in the supernatant. **k** Fold-change of differential PUFAs detected by our supernatant-targeted metabolomics. **l** Venn diagram showing the 4 shared differential PUFAs between intracellular and supernatant-targeted metabolomics. DM differentially abundant metabolites, AA arachidonic acid, DGLA dihomogamma-linolenic acid, EPA eicosapentaenoic acid, DHA docosahexaenoic acid. **m** DHA concentrations in the supernatants of PLC/PRF/5 and Huh7 cells were measured via ELISA. **n** Schematic diagram of the CD8^+^ T-cell-mediated tumor killing assay (upper panel), which was conducted by coculturing activated CD8^+^ T cells and wt PLC/PRF/5 or Huh7 cells. Before cocultivation, activated CD8^+^ T cells were pretreated with 20 μmol DHA or NC. Representative images are shown in the bottom panel. CM conditioned medium; 0.1% v/v BSA-ethanol was used as a negative control (NC) for DHA treatment. **o** Schematic diagram of the CD8^+^ T-cell-mediated tumor killing assay (upper panel), which was conducted by coculturing activated CD8^+^ T cells and wt PLC/PRF/5 or Huh7 cells. Before cocultivation, activated CD8^+^ T cells were pretreated with 20 μmol DHA or NC plus CM supernatant from PLC/PRF/5 cells transfected with vector or ovTACC3. Representative images are shown in the bottom panel. CM conditioned medium; 0.1% v/v BSA-ethanol was used as a negative control (NC) for DHA treatment. **p** Representative image and statistical chart of the subcutaneous HCC model. Hepa1‒6 cells were transfected with or without ovTACC3. The mice were given 100 mg of DHA by daily gavage. n = 6 per group. **q** Flow cytometry analysis of the subcutaneous HCC model described above. The statistical charts of the total CD8^+^ T-cell percentage, IFN-γ^+^CD8^+^ T-cell percentage, and PD-1^+^CD8^+^ T-cell percentage are shown in the right panel. n = 6 per group. The data and error bars represent the means ± SDs. The data were analyzed via Student’s t test (**e**, **f**, **k**, **m**‒**q**)

Intracellular targeted metabolomics of four shNC and three shTACC3 PLC/PRF/5 cells revealed 50 differentially abundant metabolites, with fatty acids constituting 22% of these species (Fig. 4a‒c). Notably, PUFAs, particularly the n-3 subtypes, represented more than half of the differential fatty acids (Fig. 4d). Pathway enrichment analysis further confirmed the association of TACC3 with PUFA metabolic pathways, including omega-hydroxylase activity (Supplementary Fig. 5e). Specifically, TACC3 knockdown markedly increased the intracellular levels of arachidonic acid (AA), EPA, docosapentaenoic acid (DPA), and DHA (Fig. 4e), a pattern validated by ELISA quantification of DHA (Fig. 4f and Supplementary Fig. 6a).

Given the compartmentalized nature of tumor-immune interactions, we hypothesized that TACC3 regulates PUFA secretion into the TIME. Targeted metabolomics was performed again via CM from three shNC and three shTACC3 PLC/PRF/5 cells (Fig. 4g, h), and the results revealed that 26.67% of the differentially abundant metabolites were fatty acids, 50% of which were PUFAs (Fig. 4i, j). Strikingly, the extracellular AA, EPA, and DHA concentrations increased 2.3–13.1-fold upon TACC3 knockdown (Fig. 4k), with four PUFAs identified as shared metabolites through intersection analysis (Fig. 4l). Furthermore, pathway enrichment analysis based on the predicted metabolite sets library of differentially abundant metabolites identified by supernatant-targeted metabolomics revealed that TACC3 reprograms PUFA metabolism within HCC cells, thereby altering PUFA composition in the TME (Supplementary Fig. 5f). More importantly, ELISA validation confirmed TACC3’s dual control over intracellular and extracellular PUFA pools, especially DHA (Fig. 4m and Supplementary Fig. 6b).

To explore the functional relevance of DHA, we pretreated CD8^+^ T cells with DHA prior to coculture with wild-type (wt) HCC cells. This n-3 PUFA supplementation significantly enhanced T-cell-mediated tumor cytotoxicity in vitro (Fig. 4n and Supplementary Fig. 6c) and rescued the functional impairment induced by CM from ovTACC3-expressing HCC cells (Fig. 4o and Supplementary Fig. 6d, e). In murine models, oral administration of DHA significantly attenuated the tumor growth promoted by TACC3 overexpression (Fig. 4p). Flow cytometry analysis further demonstrated that DHA restored CD8^+^ T-cell infiltration and cytotoxicity hampered by TACC3 (Fig. 4q).

To clinically validate our findings, we analyzed tissue and serum samples from 10 HCC patients. The results revealed that the serum DHA levels in the TACC3-high HCC patients were markedly lower than those in the TACC3-low patients (Supplementary Fig. 6f), which aligns with our targeted metabolomics data. Flow cytometry demonstrated that TACC3-high tumors exhibited lower infiltration of total and IFN-γ^+^CD8^+^ T cells but higher PD-1^+^CD8^+^ T-cell proportions (Supplementary Fig. 6g‒i), further confirming that low microenvironmental DHA in TACC3-high HCC may promote a cold tumor immune phenotype and inhibit the antitumor immunity of CD8^+^ T cells.

In conclusion, these findings establish that TACC3 compromises CD8^+^ T-cell-mediated antitumor immunity within the HCC TIME by reprogramming PUFA metabolism.

### Key enzyme ACSL4 mediates TACC3-driven PUFA metabolism reprogramming

**Fig. 5:**
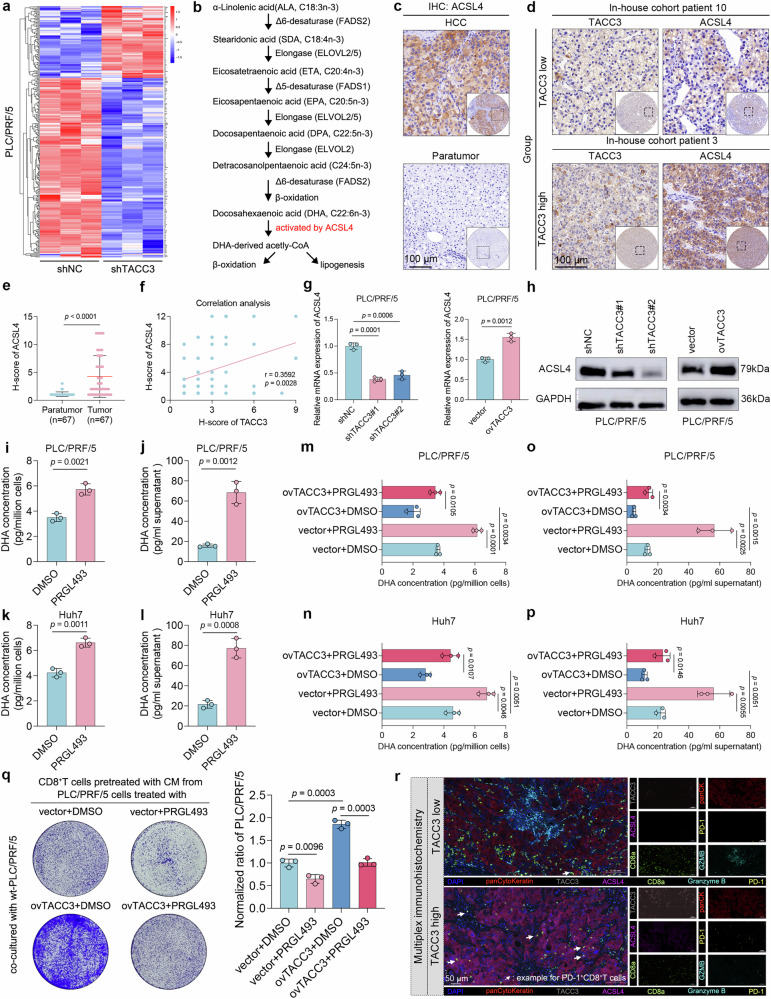
Key enzyme ACSL4 mediates TACC3-driven PUFA metabolic reprogramming. **a** Heatmap of RNA-seq data showing DEGs from 3 shNC and 3 shTACC3 PLC/PRF/5 cell lines. **b** Flow chart of n-3 PUFA metabolism. **c** ACSL4 IHC assay of a tissue microarray including 67 paired HCC tumor and peritumoral tissues from our center. Scale bars, 100 μm. **d** Correlation analysis between TACC3 and ACSL4 expression on the basis of the IHC staining intensity. Patients were divided into TACC3-high and TACC3-low groups according to the median TACC3 H score. Scale bars, 100 μm. **e** Statistical chart of the ACSL4 H score, which was determined via IHC of our tissue microarray. **f** Pearson’s correlation chart of the IHC intensity of TACC3 and ACSL4 in our tissue microarray. **g**, **h** The mRNA and protein levels of ACSL4 were detected after knockdown or overexpression of TACC3 via RT‒qPCR (**g**) and western blotting (**h**), respectively. **i, k** Intracellular DHA concentrations in PLC/PRF/5 (**i**) and Huh7 (**k**) cells treated with PRGL493 (50 μmol) were measured via ELISA. PRGL493 is a specific inhibitor of ACSL4. **j, l** DHA concentrations in the supernatants of PLC/PRF/5 (**j**) and Huh7 (**l**) cells treated with PRGL493 (50 μmol) were detected via ELISA. **m, n** Intracellular DHA concentrations of ovTACC3 PLC/PRF/5 (**m**) and ovTACC3 Huh7 (**n**) cells treated with PRGL493 (50 μmol) were measured via ELISA. **o**, **p** DHA concentrations in the supernatants of ovTACC3 PLC/PRF/5 (**o**) and ovTACC3 Huh7 (**p**) cells treated with PRGL493 (50 μmol) determined via ELISA. **q** Representative images of the CD8^+^ T-cell-mediated tumor killing assay, which was performed by coculturing activated CD8^+^ T cells and wt PLC/PRF/5 cells. Before cocultivation, activated CD8^+^ T cells were pretreated with CM from ovTACC3 PLC/PRF/5 cells treated with PRGL493 (50 μmol). The statistical chart is shown in the right panel. **r** mIHC analysis showing the colocalization of TACC3, ACSL4 and GZMB^+^CD8^+^ T cells or PD-1^+^CD8^+^ T cells. Scale bars, 50 μm. Data and error bars are presented as the means ± SDs. The data were analyzed via the Wilcoxon signed-rank test (**e**), Pearson’s correlation analysis (**f**), and Student’s t test (**g**, **i**‒**q**)

Subsequent investigations focused on elucidating the mechanism by which TACC3 regulates PUFA metabolism in HCC cells. Notably, among the metabolism-related DEGs identified in our RNA-seq analysis (Fig. 5a and Supplementary Table 2), ACSL4 has emerged as a prime candidate because of its established role in generating PUFA-derived acyl-CoA (Fig. 5b),^27–30^ and a reduction in PUFA-derived acyl-CoA and DHA-containing phospholipids was observed upon ACSL4 knockdown.^31^ Given the inverse correlation between TACC3 knockdown-induced PUFA accumulation and ACSL4 downregulation, we hypothesized that ACSL4 serves as a key mediator in TACC3-regulated PUFA metabolism reprogramming.

Consistent with the ICGC-LIHC dataset (Supplementary Fig. 7a), our tissue microarray IHC revealed significantly elevated ACSL4 expression in tumors versus adjacent normal tissues (Fig. 5c, e). Elevated ACSL4 expression was marginally associated with poor OS in HCC patients, but the difference was not statistically significant (Supplementary Fig. 7b). Strikingly, both mRNA coexpression analysis (Supplementary Fig. 7c) and protein-level IHC correlation (Fig. 5d, f) revealed a strong positive association between TACC3 and ACSL4. Mechanistically, RT‒qPCR and immunoblotting confirmed the specific regulatory effect of TACC3 on ACSL4 expression in PLC/PRF/5 and Huh7 cells (Fig. 5g, h and Supplementary Fig. 7d‒j) without affecting other PUFA pathway enzymes, such as fatty acid desaturase 1 (FADS1) (Supplementary Fig. 7k).

Next, we sought to validate whether ACSL4 is necessary for TACC3-mediated PUFA metabolic reprogramming. Functional validation through PRGL493 treatment, previously reported as a specific inhibitor of ACSL4, recapitulated the PUFA accumulation phenotype observed with TACC3 knockdown, as evidenced by increased intracellular and extracellular DHA levels (Fig. 5i‒l).^32^ Crucially, PRGL493 cotreatment partially reversed the TACC3 overexpression-induced suppression of DHA production (Fig. 5m‒p), establishing ACSL4 as a downstream effector in the TACC3-mediated PUFA metabolic pathway.

To investigate the immunological consequences of the TACC3/ACSL4/PUFA axis, we employed a coculture model in which CM from TACC3-overexpressing HCC cells decreased the cytotoxicity of CD8^+^ T lymphocytes. Notably, this immunosuppressive effect was attenuated by PRGL493 treatment of HCC cells (Fig. 5q and Supplementary Fig. 7l). Furthermore, mIHC clinical validation revealed dual correlations: positive associations between TACC3 expression and both ACSL4 expression and PD-1^+^CD8^+^ T-cell infiltration; and an inverse relationship between TACC3 levels and GZMB^+^CD8^+^ T-cell populations (Fig. 5r).

These findings collectively establish ACSL4 as the pivotal enzyme mediating TACC3-driven PUFA metabolic reprogramming and subsequent CD8^+^ T-cell dysfunction.

### TACC3 stabilizes ACSL4 mRNA by binding to LARP1 and PABPC1

Next, we tried to elucidate the mechanism underlying the TACC3-mediated regulation of ACSL4 expression. While our data indicated that TACC3 regulates ACSL4 at the mRNA level, no intrinsic RNA-binding capacity has been reported for TACC3. This prompted us to hypothesize that indirect regulation occurs through RNA-binding protein (RBP) intermediaries.

**Fig. 6:**
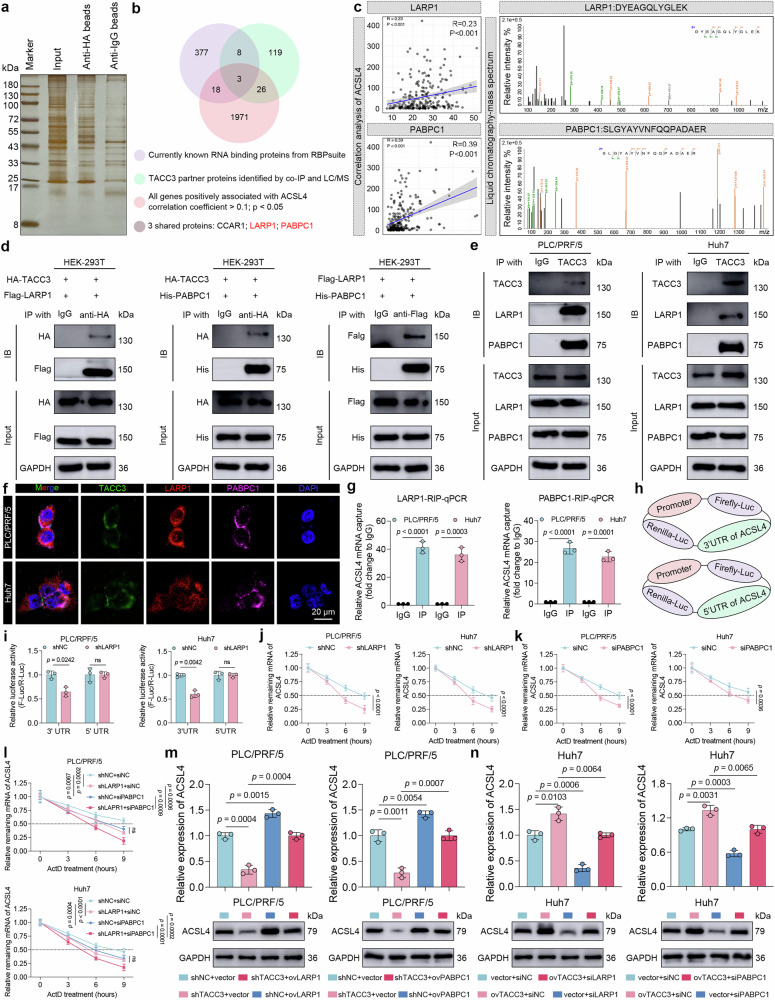
TACC3 stabilizes ACSL4 mRNA by binding to LARP1 and PABPC1. **a** A co-IP assay combined with LC‒MS was performed to detect the partner proteins of TACC3 in PLC5/PRF/5 cells transfected with HA-tagged TACC3. Co-IP coimmunoprecipitation, LC‒MS liquid chromatography‒mass spectrum. **b** Venn diagram showing how LARP1 and PABPC1 were identified as potential downstream binding proteins of TACC3. **c** Scatter plots of Pearson’s correlations showing the relationships between LARP1/PABPC1 and ACSL4 mRNA expression (left panel). The protein secondary structures of LARP1 and PABPC1, as identified by LC‒MS, are shown in the right panel. **d** Co-IP was combined with western blotting in HEK-293T cells to explore the exogenous interactions between TACC3 and LARP1, TACC3 and PABPC1, and LARP1 and PABPC1. **e** Co-IP assay combined with western blotting in PLC5/PRF/5 and Huh7 cells to confirm the endogenous binding between TACC3, LARP1, and PABPC1. **f** Immunofluorescence showing the cytoplasmic colocalization of TACC3, LARP1, and PABPC1 in PLC5/PRF/5 and Huh7 cells. Scale bars, 20 μm. **g** A RIP assay combined with RT‒qPCR analysis was conducted to detect the interaction of ACSL4 mRNA with LARP1 and PABPC1 in PLC5/PRF/5 and Huh7 cells. RIP RNA immunoprecipitation. **h** Schematic diagram of the dual-luciferase reporter plasmids. The 3’ UTR or 5’ UTR of ACSL4 mRNA was cloned downstream of the firefly luciferase gene. Renilla luciferase served as an internal control. UTR untranslated region, Luc luciferase. **i** shLARP1-transfected HCC cells were transfected with dual-luciferase plasmids as described in (**h**). A luciferase reporter gene assay was subsequently performed. **j**‒**l** The degradation rate of ACSL4 mRNA was detected via RT‒qPCR in HCC cells treated with ActD (5 μg/mL). ActD actinomycin D. **m**, **n** Rescue experiments detecting ACSL4 mRNA and protein levels were performed through RT‒qPCR and western blotting using the indicated HCC cells. Data and error bars are presented as the means ± SDs. The data were analyzed via Pearson’s correlation analysis (**c**) and Student’s t test (**g**, **i**‒**n**)

Using HA-tagged TACC3 expressed in HEK-293T cells, coimmunoprecipitation (co-IP) coupled with liquid chromatography‒mass spectrometry (LC‒MS) identified 156 candidate interactors (Fig. 6a and Supplementary Table 3). Cross-referencing these with known RBPs (Supplementary Table 4) and the ICGC-LIHC transcriptomic dataset (ACSL4-correlated genes, correlation coefficient > 0.1, *p* < 0.05; Supplementary Table 5), we identified three potential mediators (Fig. 6b, c and Supplementary Table 6). Notably, LARP1 and PABPC1 have emerged as prime candidates given the established roles of LARP1 and the auxiliary function of PABPC1 in 3’ untranslated region (UTR)-mediated mRNA stabilization,^33,34^ elevated HCC expression patterns (Supplementary Fig. 8a, b), and documented oncogenic functions.^35,36^

Co-IP experiments in HEK293T cells confirmed reciprocal binding among TACC3, LARP1, and PABPC1 (Fig. 6d). Endogenous interactions were validated within PLC/PRF/5 and Huh7 cells (Fig. 6e), with cytoplasmic colocalization observed via immunofluorescence (Fig. 6f).

RNA immunoprecipitation (RIP) assays demonstrated significant enrichment of ACSL4 mRNA by LARP1 and PABPC1 antibodies compared with IgG controls (Fig. 6g). In addition, after interfering with TACC3 expression, the binding of LARP1 and PABPC1 to ACSL4 mRNA was significantly reduced, and there was no change in the expression levels of the two proteins (Supplementary Fig. 8c‒f), indicating that the presence of TACC3 provides conditions that are conducive to binding. We further explored whether LARP1 binds to the 3’ UTR or 5’ UTR of ACSL4 mRNA. Luciferase reporter assays demonstrated specific binding of LARP1 to the 3’ UTR but not the 5’ UTR of ACSL4. This interaction was validated by shRNA-mediated LARP1 knockdown, which selectively attenuated the 3’ UTR luciferase signal while leaving the 5’ UTR activity unaffected (Fig. 6h, i). Similarly, an RNA pull-down assay also revealed that LARP1 interacted with the 3’ UTR of ACSL4 mRNA but not the 5’ UTR (Supplementary Fig. 8g). To delineate the specific region governing LARP1-ACSL4 mRNA interactions, we performed a comprehensive mutational analysis of the La motif (LaM) domain, which was previously reported as a conserved RNA-binding module containing three critical aromatic/hydrophobic residues (Q333, Y336, and F348) (Supplementary Fig. 8h).^37^ Site-directed mutagenesis of individual residues (Q333A, Y336A, and F348A) followed by RNA pull-down assays revealed preserved binding capacity. However, combinatorial double mutations (Q333A/F348A, Q333A/Y336A, and F348A/Y336A) completely abrogated LARP1-ACSL4 mRNA complex formation (Supplementary Fig. 8i), demonstrating the indispensability of the LaM region in maintaining RNA interactions. We subsequently identified two AU-rich elements (AREs) (ARE1: 3530‒3536 nt, ARE1: 4304‒4310 nt) in ACSL4’s 3’ UTR, as AREs are the canonical targets of RBPs (Supplementary Fig. 8j). RNA pull-down assays employing ARE-mutant probes demonstrated that single ARE mutations reduced but did not completely block LARP1 recruitment, whereas dual mutations abolished this binding (Supplementary Fig. 8k). Thus, our data indicate that the LaM region of LARP1 recognizes AREs in the 3’ UTR and interacts with ACSL4 mRNA.

Given the canonical role of RBPs in mRNA stabilization,^38,39^ we assessed ACSL4 transcript stability via the use of actinomycin D, which blocks de novo RNA synthesis, as previously reported.^40,41^ Interference with LARP1 or PABPC1 accelerated ACSL4 mRNA degradation (Fig. 6j, k), with combined knockdown resulting in a more significant decrease (Fig. 6l). Rescue experiments confirmed the regulatory effect of TACC3 on these RBPs, as LARP1 or PABPC1 silencing attenuated TACC3 overexpression-induced ACSL4 upregulation (Fig. 6m, n and Supplementary Fig. 6l).

Collectively, our findings suggest that LARP1 and PABPC1 stabilize ACSL4 mRNA through 3’ UTR binding. TACC3 then specifically upregulates ACSL4 mRNA by functionally interacting with these two RBPs.

### GalNAc–siTACC3 synergizes with PD-1 blockade to enhance antitumor immunity

To determine the immunoregulatory role of TACC3, we explored whether targeting TACC3 could enhance the efficacy of ICIs. For liver targets, several studies have demonstrated that N-acetylgalactosamine (GalNAc) is a well-established delivery vehicle due to its high affinity for the asialoglycoprotein receptor (ASGR), which especially exhibits preferential membrane localization in hepatocytes.^42,43^ Punit P. Seth et al. reported that the effectiveness of antisense oligonucleotides increases by 6–10-fold with GalNAc conjugation.^44^ The US Food and Drug Administration (FDA) has approved GalNAc-conjugated siRNA (GalNAc–siRNA) therapeutics for multiple disorders, including acute porphyria, transthyretin amyloidosis, hypercholesterolemia, and primary hyperoxaluria type 1.^45^ Emerging candidates such as RG4346 (for chronic hepatitis B virus infection) further demonstrated the platform’s clinical viability through ongoing trials.^46^

**Fig. 7:**
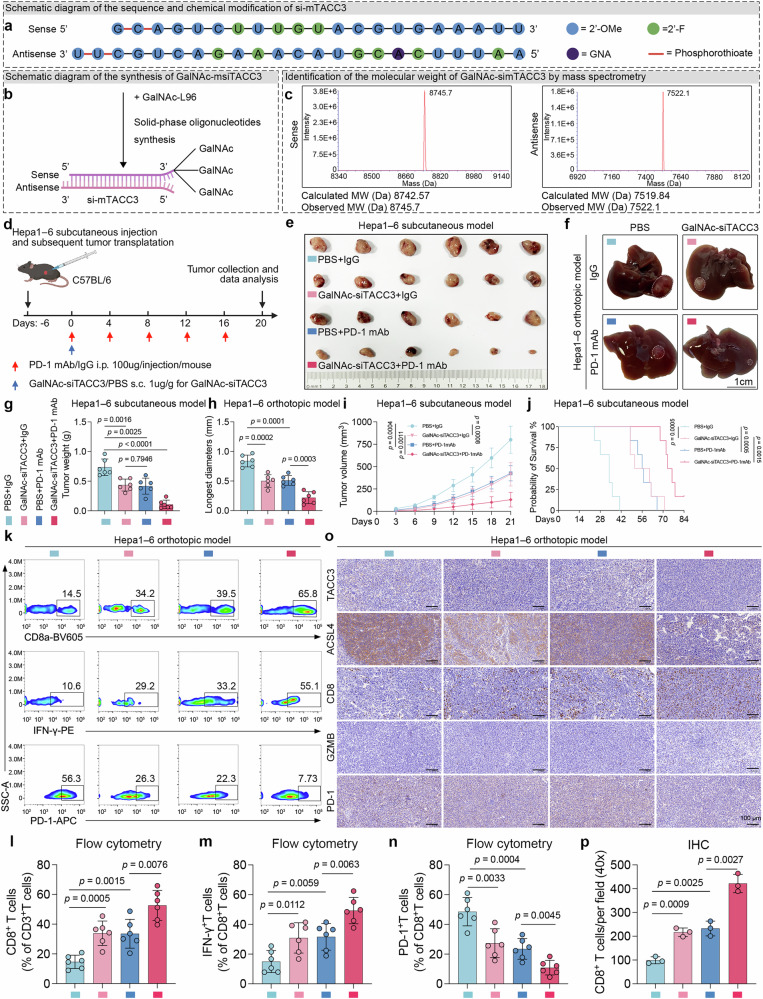
GalNAc–siTACC3 synergizes with PD-1 blockade to enhance antitumor immunity. **a**‒**c** The graphic design of the construction process of GalNAc–siTACC3. Sequence and chemical modifications of si-mTACC3 (**a**). Synthesis of GalNAc–siTACC3 via a reaction between GalNAc-L96 and si-mTACC3 (**b**). Identification of GalNAc–siTACC3 by mass spectrometry (**c**). 2’-OMe 2’-O-Methyl, 2’-F 2’-Fluoro, GNA glycol nucleic acid. mTACC3 mouse TACC3. **d** Schematic diagram of the animal models and drug administration, including the PD-1 mAb and GalNAc–siTACC3. s.c. subcutaneous injection. **e**, **f** Representative images of the subcutaneous (**e**) and orthotopic (**f**) tumor models. Scale bars for the orthotopic tumor, 1 cm. **g**‒**j** Statistical charts of weight (**g**), growth curves (**i**), and survival curves (**j**) of the subcutaneous tumor models and the longest diameter of the orthotopic tumors (**h**). **k**‒**n** Flow cytometry analysis of orthotopic tumors (**k**) and statistical plots of total CD8^+^ T cells (**l**), IFN-γ^+^CD8^+^ T cells (**m**), and PD-1^+^CD8^+^ T cells (**n**). n = 6 per group. **o, p** IHC experiments of TACC3, ACSL4, CD8, GZMB, and PD-1 in orthotopic tumors (**o**) and a statistical analysis of infiltrating CD8^+^ T cells (**p**). Scale bars, 100 μm. Data and error bars are presented as the means ± SDs. The data were analyzed via Student’s t test (**g**, **h**, **i**, **l**‒**p**) and the log-rank (Mantel‒Cox) test (**j**)

Motivated by these findings, we sought to develop GalNAc–siTACC3 as a synergistic treatment with an anti-PD-1 mAb to improve HCC tolerance to current immunotherapies. Four siRNAs targeting mouse tacc3 were designed, and their silencing efficacy was validated via RT‒qPCR in Hepa1‒6 cells (Supplementary Fig. 9a). As illustrated in the schematic diagram (Fig. 7a, b), GalNAc–siTACC3 was constructed by coupling GalNAc with base-modified si-tacc3#3, which exhibited the highest silencing efficacy against tacc3. The molecular weight of GalNAc–siTACC3 was confirmed by mass spectrometry, and the results were consistent with the expected calculated molecular weight (Fig. 7c).

The therapeutic safety profile of GalNAc–siTACC3 was comprehensively assessed through integrated in vitro and in vivo evaluations. In vitro CCK-8 assays confirmed that the viability of nontumorigenic THLE-2 human hepatic epithelial cells was maintained (Supplementary Fig. 9b). In vivo safety evaluation via subcutaneous administration in healthy C57BL/6 mice revealed serum alanine aminotransferase (ALT)/aspartate aminotransferase (AST) levels within normal physiological ranges (Supplementary Fig. 9c) and demonstrated undamaged multiorgan architecture through histopathological analysis (Supplementary Fig. 9d), collectively indicating systemic biocompatibility. Mechanistic validation of hepatic targeting revealed two critical findings. First, RT‒qPCR analysis verified the absence of significant TACC3 mRNA suppression in THLE-2 cells (Supplementary Fig. 9e). Second, Cy3-labeled GalNAc–siTACC3 exhibited exclusive hepatic fluorescence within 4 hours postadministration, confirming liver-selective accumulation (Supplementary Fig. 9f). This phenomenon corresponds to relatively low ASGR and TACC3 baseline expression patterns in quiescent liver tissue.^47^ In an orthotopic HCC model, regular monitoring revealed sustained therapeutic efficacy characterized by rapid and durable TACC3 mRNA suppression without transcriptional rebound (Supplementary Fig. 9g, h). This pharmacokinetic persistence is consistent with the reported stability of GalNAc–siRNA drugs,^48^ supporting their biological feasibility as combination immunotherapy candidates.

To exclude potential off-target effects. CCK-8 assays confirmed that GalNAc–siTACC3 failed to suppress tumor cell viability in TACC3-knockout Hepa1‒6 cells (Supplementary Fig. 9i), establishing its dependence on intact TACC3 expression. To extend these findings in vivo, we established subcutaneous tumors in both nude and C57BL/6 mice via the use of sgTACC3 Hepa1‒6 cells. Importantly, the therapeutic efficacy of GalNAc–siTACC3 was nearly abolished in sgTACC3 tumors compared with that of GalNAc–siNC (Supplementary Fig. 9j‒m). Collectively, these in vitro and in vivo results provide mechanistic validation that GalNAc–siTACC3 exerts antineoplastic effects exclusively through TACC3 suppression.

The potential of combination therapy with PD-1 blockade and GalNAc–siTACC3 was further validated in preclinical murine models (Fig. 7d). Compared with those of the groups treated with GalNAc–siTACC3 or PD-1 mAb alone, the results of both the subcutaneous and orthotopic tumor models indicated that GalNAc–siTACC3 and PD-1 mAb strikingly inhibited tumor growth (Fig. 7e‒i) and improved OS time (Fig. 7j). Furthermore, RT‒qPCR analysis of orthotopic tumors revealed that GalNAc–siTACC3 indeed significantly inhibited TACC3 expression (Supplementary Fig. 9n).

Next, flow cytometry (Fig. 7k) and IHC analysis (Fig. 7o) revealed that, compared with PD-1 mAb monotherapy, combination therapy with GalNAc–siTACC3 plus an anti-PD-1 mAb induced significant remodeling of the TIME. The immunologically inert “cold” tumor phenotype transitioned to an inflamed “hot” tumor state, characterized by increased infiltration of total CD8^+^ T lymphocytes (Fig. 7l, p and Supplementary Fig. 9p). Quantitative assessments revealed a marked increase in the ratio of IFN-γ-producing CD8^+^ T lymphocytes (Fig. 7m) and an obvious reduction in exhausted PD-1^+^CD8^+^ T-cell populations (Fig. 7n). IHC profiling further confirmed the therapeutic efficacy of GalNAc–siTACC3, which markedly downregulated both TACC3 and ACSL4 protein expression in treated samples (Fig. 7o and Supplementary Fig. 9o).

To summarize, these findings substantiate targeting TACC3 as a feasible strategy for TIME remodeling in HCC, potentially overcoming the current limitations of ICI resistance.

## Discussion

HCC continues to rank among the top causes of tumor-associated mortality worldwide, and its five-year OS rate has long stagnated below 20%.^49^ Recently, immunotherapies, particularly PD-1/PD-L1 inhibitors, have made significant breakthroughs in extending the OS time of a certain subset of HCC patients.^50^ However, the <20% ORR of monoimmunotherapy underscores the critical need for more predictive biomarkers and novel combinatorial strategies.^51^ Through cross-model multiomics integration, we discovered that TACC3 drives CD8^+^ T-cell dysfunction via PUFA metabolic remodeling, establishing a previously unreported TACC3-LARP1/PABPC1-ACSL4-PUFA metabolism axis that fundamentally reshapes the TIME. Our findings may provide a new treatment strategy for HCC immunotherapy resistance.

While previous studies have established the role of TACC3 in mitotic regulation, tumor proliferation, and chemoresistance, our findings extend this understanding by revealing its novel immunosuppressive functions in HCC.^13,14,16,17^ Building on the reported association between TACC3 and immunogenic cell death in BRCA, we employed multimodal validation through animal models, scRNA-seq, and complementary in vitro and in vivo assays to demonstrate TACC3-mediated immune modulation in HCC.^15^ Our data corroborate its proliferative role while newly identifying its capacity to reshape the TIME through lipid metabolic reprogramming. This metabolic shift appears to induce TIME polarization from immunologically active “hot” to suppressive “cold” phenotypes, suggesting a mechanistic link between tumorous TACC3-driven metabolic alterations and immune evasion. Increasing evidence indicates that tumor progression is fuelled by lipid metabolic reprogramming, which provides energy substrates, signaling mediators, and membrane components, thereby facilitating crosstalk with other cells in the TIME.^52^ Our integrated RNA sequencing and metabolomic analyses of HCC cells revealed that TACC3 predominantly modulates n-3 PUFA metabolism. Intriguingly, while n-6 PUFA derivatives such as LA enhance cytotoxic T lymphocyte function through mitochondrial optimization, the immunoregulatory function of tumor-derived n-3 PUFA metabolites remains poorly understood.^8^ Our key findings demonstrate that DHA, the terminal metabolite of the n-3 PUFA pathway, potentiates CD8^+^ T-cell-mediated tumor cytotoxicity. This finding aligns with clinical observations correlating increased dietary DHA intake with reduced colorectal cancer risk in patients with high CD8^+^ T-cell infiltration.^53^ Remarkably, DHA supplementation restored antitumor efficacy in CD8^+^ T lymphocytes preexposed to CM from TACC3-overexpressing HCC cells, establishing TACC3-driven metabolic rewiring as a mechanism of immune evasion through manipulation of the n-3 PUFA pathway.

While our findings establish DHA as a potent enhancer of CD8^+^ T-cell-mediated antitumor immunity, the molecular circuitry governing this immunomodulatory phenomenon remains incompletely mapped. Currently, some studies indicate that the increased activity of tumor-infiltrating CD8^+^ T cells is related to mechanisms such as the activation of specific signaling pathways, improved metabolism, and reorganization of the cell structure. For example, the mTOR pathway emerges as a critical regulatory nexus, coordinating both the decision of CD8^+^ T-cell destiny (naive T-cell to effector/memory T-cell transition) and bioenergetic adaptation through tricarboxylic acid cycle (TCA) modulation during activation.^54–56^ In addition, a previous study demonstrated that DHA induces lipid remodeling of the cancer cell membrane, enhancing fluidity while obstructing the PD-L1/PD-1 interaction,^57^ which may also explain why a high level of DHA is associated with an increased number of total and effector CD8^+^ T cells, as observed in our research. Nevertheless, such tumor-related mechanisms fail to account for the cell-intrinsic immunostimulatory effects of DHA on CD8^+^ T cells. Complementary work by Teresa Manzo et al. revealed that LA reprograms CD8^+^ T-cell lipidomes through stimulating lipid biosynthesis and optimizing mitochondrial respiration.^8^ However, critical uncertainties persist regarding whether DHA performs analogous membrane reorganization to influence the distribution of immune checkpoints or coordinates metabolic reprogramming to sustain effector functions in CD8^+^ T cells. To disentangle these mechanistic possibilities, attention should be focused on bioenergetic substrate provisions, such as acetyl-CoA, the activation of key signaling pathways associated with nutrition and proliferation, and membrane domain restructuring, which impacts surface receptor clustering. Systematic interrogation of these noncompeting mechanisms will clarify how n-3 PUFAs functionally sustain the antitumor immunity mediated by CD8^+^ T cells within the TIME.

In the context of the established molecular mechanisms of TACC3-dependent tumorigenesis, therapeutic development has progressed to develop small-molecule inhibitors that target TACC3.^58,59^ While these agents demonstrate the potential of TACC3 as an immunotherapeutic adjuvant, their clinical utility remains constrained by off-target effects and acquired resistance. To circumvent these limitations in HCC immune checkpoint blockade, we employed RNA interference (RNAi) technology for TACC3 modulation. However, the inherent difficulty in the targeted delivery of RNAi therapeutics is a key challenge. Recent advances in hepatic targeting strategies show particular promise, with GalNAc–siRNA formulations demonstrating both hepatotropism and clinical safety. This is exemplified by RG6346, a GalNAc-encapsulated RNAi agent against chronic hepatitis B virus (HBV) infection that has achieved favorable safety profiles and antiviral efficacy in phase I trials. RG6346 (four 4-week courses) suppresses HBV infection for up to 392 days, findings that are corroborated by our supplementary data.^46^ Additionally, mature chemical modifications, including 2’-O-methyl (2’-OMe), 2’-fluoro (2’-F), and phosphorothioate linkages, enhance siRNA stability in vivo. Studies have confirmed that terminal phosphorothioate modifications significantly improve the antinuclease activity of GalNAc–siRNA conjugates and prolong their gene silencing duration.^48^ Our siRNA design incorporates these optimizations, specifically integrating 2’-OMe, 2’-F, and phosphorothioate bonds. Leveraging this delivery platform, we engineered GalNAc–siTACC3, with integrated 2’-OMe, 2’-F, and phosphorothioate modifications—critical stabilizers of siRNA therapeutics—to potentiate PD-1 blockade therapy. Our results demonstrate that combinatorial GalNAc–siTACC3/PD-1 mAb treatment outperforms monotherapeutic efficacy through two mechanisms: enhanced tumor growth suppression and microenvironmental reprogramming, which converts immunologically “cold” tumors to “hot” phenotypes. These findings position TACC3 as a compelling adjunctive target for overcoming immunotherapy resistance in HCC.

**Fig. 8:**
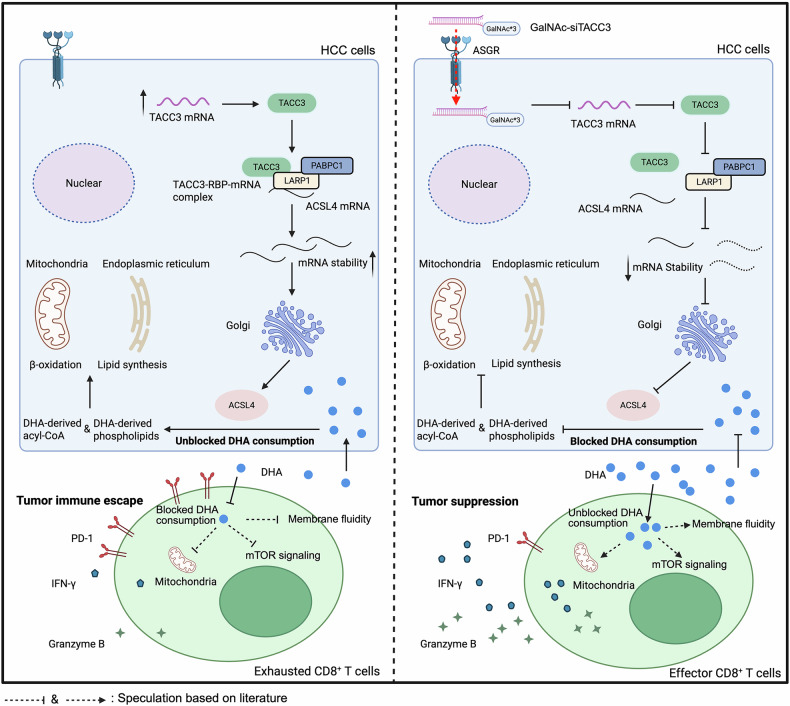
Schematic diagram depicting that TACC3-mediated PUFA metabolic reprogramming via the LARP1/PABPC1-ACSL4 axis reduces DHA levels in the TME, thereby suppressing CD8^+^ T-cell antitumor immunity. Therapeutic targeting of TACC3 may increase the efficacy of HCC immunotherapy. The schematic diagram was created using BioRender and included a publication license (Chen (2025) https://BioRender.com/dsxgsmu)

In conclusion, elevated TACC3 expression in ICI-nonresponsive HCC patients is correlated with diminished CD8^+^ T-cell cytotoxicity and unfavorable clinical outcomes. TACC3 orchestrates a LARP1/PABPC1 RNA-binding complex to stabilize ACSL4 transcripts, driving PUFA metabolic remodeling that depletes DHA in the TME. This lipidomic perturbation critically impairs CD8^+^ T-cell-mediated tumor elimination, fostering immunotherapy resistance (Fig. 8). Our therapeutic investigation revealed that GalNAc-conjugated siTACC3 synergizes with PD-1 blockade to overcome treatment resistance, while clinical translation analysis suggested that dietary DHA supplementation is a viable adjuvant strategy. Overall, our findings establish TACC3 as both a prognostic biomarker and therapeutic target to improve HCC immunotherapy efficacy.

## Materials and methods

### Clinical samples

This study utilized 67 paired cancer and paratumor normal tissue samples collected from HCC patients diagnosed through histopathological examination at Huashan Hospital, Fudan University, between April 2015 and January 2020. These tissues were used to construct tissue microarrays for subsequent immunohistochemical analysis. Additionally, ultrasound-guided biopsy samples were obtained from 10 nonresponders and 10 responders who received immunotherapy between August 2022 and March 2025 to validate the DEGs identified through bioinformatics analysis. Immunotherapy response was evaluated according to the Response Evaluation Criteria in Solid Tumors (RECIST) v1.1 guidelines.^60^ The study protocol was approved by the Ethics Committee of Huashan Hospital (KY2023-594), with written informed consent obtained from all participants prior to specimen collection.

### Animal studies

All animal experiments were carried out in compliance with the guidelines established by the Institutional Animal Care and Use Committee of Fudan University (2024-HSYY-315). Specific pathogen-free (SPF) animals were obtained from GemPharmatech (Jiangsu, China) and maintained under environmentally controlled conditions with a 12/12-hour light‒dark cycle at 22 ± 2 °C and a relative humidity of 50 ± 10%. The mice were randomly assigned via a computer-generated randomization method prior to treatment and experiments, with each group consisting of 6 mice to account for the variability of the in vivo experiments.

For the subcutaneous tumor model, human and mouse HCC cells were administered via subcutaneous injection into the right flank of BALB/c mice (1×10^6^/100 μL of PBS) and C57BL/6 mice (2×10^6^/100 μL of PBS), respectively. Tumor dimensions were assessed with calipers, and tumor volume was evaluated via the following formula: volume = (length × width²)/2.

Subcutaneous tumor models were established in nude mice and C57BL/6 mice via wt human or mouse HCC cells. Intratumoral siRNA injections were started on day 7 after tumor implantation. Transfection complexes were prepared as follows: scrambled RNA or siTACC3 was mixed with in vivo jetPEI transfection reagent (Polyplus-transfection, New York, NY) at an N/P ratio of 10, and each dose contained 12 μg of siRNA dissolved in 100 μL of sterile PBS. Intratumoral injections were performed every 72 hours.

For the orthotopic tumor model, minced tumor tissues derived from the subcutaneous model were implanted into the left lobe of the mouse livers.

For CD8^+^ T-cell depletion in the animal models, a mouse anti-CD8 mAb (100 μg/100 μL of PBS, BE0146, BioXcell, USA) and an isotype control antibody (100 μg/100 μL of PBS, BE0089, BioXcell, USA) were delivered through intraperitoneal injection to each group of C57BL/6 mice (n = 6 per group) on days 0, 4, 8, 12, and 16 to eliminate CD8^+^ T cells.

The health and welfare of the mice were monitored daily, and the maximum tumor weight did not exceed 10% of the animal’s body weight. When necessary, euthanasia was performed in certain cases, such as anticipated death, extreme physiological weakness, or when the tumor burden resulted in ulceration and behavioral abnormalities. The mice were anesthetized with isoflurane and euthanized by cervical dislocation.

### Targeted metabolomics

To explore the intracellular metabolites regulated by TACC3 and their impact on the microenvironment, we performed targeted metabolomics sequencing using PLC/PRF/5 cell lines (shNC, n = 4; shTACC3, n = 3; one shTACC3 sample excluded for failed quality control) with corresponding culture supernatants (shNC, n = 3; shTACC3, n = 3). Targeted metabolomics sequencing and subsequent bioinformatics analysis were conducted by Cosmos Wisdom Biotechnology Co., Ltd. (Hangzhou, China).

### CD8^+^ T-cell-mediated tumor killing assay

PBMCs were isolated from healthy donors and cultured in CTS™ AIM V™ SFM (0870112DK, Gibco, USA) supplemented with recombinant human IL-2 (1000 U/ml, R&D Systems, USA) and an ImmunoCult™ human CD3/CD28/CD2 T-cell activator (10970, STEMCELL Technologies, Canada) for seven days to obtain activated T cells. In subsequent experiments, these T cells continued to receive anti-CD3 antibody (100 ng/ml) and recombinant human IL-2 (1000 U/ml). Before the coculture of T lymphocytes and HCC cells, T lymphocytes were pretreated with the CM of the indicated HCC cells. Next, the wt PLC/PRF/5 and Huh7 cell lines were digested, resuspended, and inoculated into 12-well plates (3513, Corning, USA) for overnight incubation. Transwell chambers with a pore diameter of 0.4 μm (3401, Corning, USA) were then placed in the plates, and activated T cells were seeded into the upper chamber at a ratio of 1:3 (HCC cells: T cells) and cocultured for 48 hours. Afterward, the HCC cells in the lower chamber were rinsed, and the remaining cells were stained with crystal violet. The absorbance at 570 nm was determined via a spectrophotometer to evaluate the cytotoxic activity of the T lymphocytes.

### Flow cytometry

Samples for flow cytometry were obtained from tumor-bearing model mice and placed in OMICS-Guard Sample Preservation Buffer (570911, BD Biosciences, USA). The tumors were minced in digestion solution containing collagenase (C5138, ‌Sigma‒Aldrich, USA), DNase type IV (D5025, Sigma‒Aldrich, USA), hyaluronidase type V (H3884, Sigma‒Aldrich, USA) and Hank’s solution (abs9257, Absin, China) to obtain a single-cell suspension, which was percolated through 70 μm nylon strainers (352350, Falcon, Becton Dickinson, USA). For extracellular staining, a mixed antibody panel was incorporated into the cell suspension at appropriate dilutions, reacted for half an hour at room temperature, and shielded from light. To stain the intracellular targets, a Fixation/Permeabilization Kit (554714, BD Biosciences, New Jersey, USA) was used to permeabilize the membrane. The corresponding intracellular fluorescent dyes were then added, and the cells were maintained for half an hour at 4°C in the dark. The samples were analyzed via a flow cytometer (Beckman Coulter, USA), and the data were processed with FlowJo V10.4 (FlowJo LLC, USA). The gating tactics are shown in Supplementary Fig. 10. Information on the antibodies used for flow cytometry is listed in the Supplementary Table 8 of the Supplementary Materials.

### Synthesis of GalNAc-conjugated siTACC3

Transheep (Transheep Shanghai Biotech Co., Ltd.) custom designed and generated four unmodified siRNAs targeting mouse TACC3 mRNA. The siRNA with the highest silencing efficiency was selected for chemical modification to increase the in vivo stability. To achieve efficient liver cell-targeted delivery, we attached the GalNAc ligand to the 3’ end of the sense strand of siTACC3 through solid-phase oligonucleotide synthesis. Quality control was conducted via mass spectrometry to determine the molecular weight of GalNAc–siTACC3. GalNAc–siTACC3 was diluted with PBS and administered by subcutaneous injection at a dose of 1 μg/g. The sequences of the siRNAs used to target mouse TACC3 are listed in the Supplementary Table 7. The dosing intervals are shown in Fig. 7d.

### Statistical analysis

Statistical evaluation was performed via GraphPad Prism (version 9, GraphPad, Inc., USA) and SPSS (version 23.0, IBM Corp., USA). The data are presented as the means ± standard deviations (SDs). For two-group comparisons, a two-tailed Student’s t test was applied. For comparisons involving three or more groups, one-way ANOVA was used. Pearson’s correlation coefficient was determined to evaluate the relationship between two variables. Survival analysis was conducted via Kaplan‒Meier curves, with log-rank tests used to assess differences. A p value of less than 0.05 was considered statistically significant. Details regarding all the statistical tests, sample sizes (n), and measures of central tendency and dispersion are provided in the corresponding figure legends.

## Supplementary information

Supplementary material

Supplementary material

## Data Availability

The datasets supporting the conclusions of this article are included within the article and its additional files. The raw sequence data of RNA-seq, scRNA-seq, and targeted metabolomics reported in this paper have been deposited in the Genome Sequence Archive (Genomics, Proteomics & Bioinformatics 2021) in the National Genomics Data Center (Nucleic Acids Res 2022), China National Center for Bioinformation/Beijing Institute of Genomics, Chinese Academy of Sciences (RNA-seq: HRA012239, scRNA-seq: CRA027652, and targeted metabolomics: OMIX010886) that are publicly accessible at https://ngdc.cncb.ac.cn/.^61–63^
