# Supplementary material for: Transforming acidic coiled-coil-containing protein 3-mediated lipid metabolism reprogramming impairs CD8+ T-cell cytotoxicity in hepatocellular carcinoma
Source: Signal Transduct Target Ther. 2025 Aug 28;10:274. doi: 10.1038/s41392-025-02367-9 (PMC12391444; doi:10.1038/s41392-025-02367-9)
Supplement: Supplementary file 1 — Supplementary material [file 41392_2025_2367_MOESM1_ESM.docx]

Supplementary Materials for

**Transforming acidic coiled-coil-containing protein 3-mediated lipid metabolism reprogramming impairs CD8^+^ T-cell cytotoxicity in hepatocellular carcinoma**

Ying Li^1, 2, 3#^, Zule Chen^1^, Dongdong Wang^4^, Wei Du^5^, Ningqi Zhu^1, 2, 3^, Xiaotian Shen^1^, Xiang Mao^1^, Yinghan Su^2, 3^, Lunxiu Qin^2, 3*^, Diyu Chen^1*^, Huliang Jia^1, 2, 3*^

*Correspondence to: Lunxiu Qin (qinlx@fudan.edu.cn), Diyu Chen (21618112@zju.edu.cn) or Huliang Jia (jbl-1@163.com)

**This PDF file includes:**

Materials and Methods

Supplementary Fig. 1**‒**10 and their legends

**Other Supplementary Materials for this manuscript include the following:**

Nine additional supplementary tables (Supplementary Table 1**‒**9) are provided as separate files due to their detailed content.

Supplementary Table 1: Datasets used for target gene screening in Fig. 1a, b.

Supplementary Table 2: DEGs of our RNA-seq using 3 shNC vs 3 shTACC3 PLC/PRF/5 cells.

Supplementary Table 3: Specific partner proteins of TACC3 identified by LC‒MS.

Supplementary Table 4: Currently known RNA binding proteins from RBPsuite (http://www.csbio.sjtu.edu.cn/bioinf/RBPsuite/).

Supplementary Table 5: All genes positively associated with ACSL4 abs (correlation coefficient) > 0.1; p ≤ 0.05.

Supplementary Table 6: Shared genes of datasets mentioned in Supplementary Table 3-5.

Supplementary Table 7: Sequence of siRNA and RT‒qPCR primers used in this study.

Supplementary Table 8: Information of antibodies used in this study.

Supplementary Table 9: Genesets for calculation of the infiltration score of immune cells by ssGSEA.

Materials and Methods

Data mining and bioinformatics analysis

The expression profile data of liver cancer patients analyzed in this study comprised the TCGA-LIHC dataset and multiple GEO datasets (GSE14520, GSE36376, GSE25097, GSE54236, GSE36411, GSE64041, GSE98620), which were downloaded respectively from the TCGA database (https://xenabrowser.net/datapages/) and GEO repository (https://www.ncbi.nlm.nih.gov/geo/) respectively. Raw data were log2-transformed after adding a pseudocount of 1, normalized using the limma package, and annotated with gene symbols through Ensembl identifiers and the human genome reference file corresponding to each dataset. DEGs were identified employing the DESeq2 algorithm for RNA-seq data (TCGA) and the limma algorithm for microarray data (GEO).

Functional enrichment analysis of DEGs was processed via the clusterProfiler R package, with gene ontology (GO) terms and KEGG pathways considered significantly enriched at a Benjamini-Hochberg adjusted *p*-value < 0.05. Visualization of enrichment results was generated using the enrichplot package. For immune cell infiltration quantification, ssGSEA was carried out with the GSVA package, utilizing the TCGA-LIHC dataset and a published immune cell marker gene list (Supplementary table 9).

The prognostic gene set for liver cancer patients was downloaded from the Human Protein Atlas (https://www.proteinatlas.org/about/download/). Genes co-expressed with ACSL4 were screened using the Pearson correlation algorithm, with a threshold of R > 0.1 and *p* < 0.05 applied during the screening process. RNA-binding proteins (RBP) were retrieved from RBPsuite (http://www.csbio.sjtu.edu.cn/bioinf/RBPsuite/). Survival data for TACC3 and its correlation with Ki67 and immune factors such as PD-L1 were evaluated through online platforms: GEPIA (http://gepia.cancer-pku.cn/index.html), KMplot (https://kmplot.com/analysis/), TISIDB (http://cis.hku.hk/TISIDB/index.php), and TIMER2.0 (http://timer.comp-genomics.org/timer/).

Cell lines

The Hepa1-6 murine hepatocellular carcinoma cell line, along with human liver cancer lines PLC/PRF/5, HuH-7, THLE-2, and HEK-293T cells were sourced from the Chinese Academy of Sciences’ Institute of Biochemistry and Cell Biology (Shanghai). All cell cultures were maintained in DMEM (Dulbecco’s Modified Eagle Medium) containing 10% FBS (F8318, Sigma-Aldrich, USA) and 1% penicillin-streptomycin (15140-122, Gibco, USA). Cell propagation was conducted under standard culture conditions: 37°C in a humidified atmosphere with 5% CO_2_. Routine mycoplasma testing was conducted to ensure that the cells were free from contamination prior to proceeding with further experiments.

Transient Transfection and Stable Cell Line Establishment

Lentiviral vectors encoding TACC3 overexpression construct, shRNA-targeting TACC3 (shTACC3), shLARP1, and PABPC1 overexpression construct were supplied by Genechem (Genechem Co., Ltd., Shanghai, China), and CRISPR/Cas9 sgRNA plasmids targeting TACC3 (sgTACC3) was purchased from Tsingke Biotechnology (Beijing, China). Viral particles were packaged using a third-generation lentiviral system (pMD.2G, psPAX2) with PEI transfection. HCC cells were infected with lentivirus at an optimized multiplicity of infection (MOI) in a serum-free medium containing 8 μg/ml polybrene (#TR-1003, Sigma-Aldrich, USA). We replaced the medium with a complete medium after incubating for approximately 12 hours. Stable pools were selected with 2 μg/ml puromycin (A1113803, Thermo) until the complete death of non-transduced controls (5-7 days), with daily medium replacement. siRNAs targeting TACC3, LARP1, and PABPC1 (Genechem Co., Ltd., Shanghai, China) were introduced using Lipofectamine 3000 (Thermo Fisher Scientific, MA, USA) as described previously. Empty vectors and scrambled siRNA were used as negative controls. Transfection efficiency was confirmed by western blotting and RT‒qPCR, with comparison to negative controls.

CCK-8 assay

Harvest exponentially growing cells and prepare a single-cell suspension in a complete medium. Seed cells in 96-well plates at 1-5 × 10³ cells/well in 100 μL medium (optimize density based on proliferation rate), followed by gentle swirling of plates to ensure uniform distribution. After 24 hr incubation at 37°C (with >90% cellular adherence confirmed by inverted microscopy), 10 μL CCK-8 solution (C0039, Beyotime) was added to each well, including blank control wells containing medium and CCK-8 without cells. Plates were incubated at 37°C for a pre-optimized duration (1-4 hr, predetermined by kinetic assays to ensure OD450nm measurements remained within linear range and below 2.0). Following gentle agitation to homogenize formazan distribution. Measure the absorbance at 450 nm using a microplate reader.

RNA-seq

We performed RNA sequencing using 3 shNC and 3 shTACC3 PLC/PRF/5 cells. The sequencing was conducted by Tsingke Biotechnology (Beijing, China).

RNA extraction and quantitative real-time PCR (RT‒qPCR)

Following the manufacturer’s guidelines, total RNA from mouse tissues and HCC cells was isolated employing the RNAeasy™ Animal RNA Isolation Kit (R0027, Beyotime, China), with RNA integrity confirmed by 1.5% agarose gel electrophoresis (28S/18S ratio >1.8) and purity assessed by Nanodrop 2000 (A260/A280 = 1.8-2.0) (Thermo Fisher Scientific, MA, USA). Following DNase I treatment during extraction, reverse transcription to cDNA was performed using the Hifair III 1st Strand cDNA Synthesis SuperMix Kit (gDNA digester plus) (11141ES60, YEASEN, China) for the following qPCR. Next, RT‒qPCR was conducted using the Hieff qPCR SYBR Green Master Mix Kit with low Rox plus (11202ES08, YEASEN, China) and the ABI PRISM 7900HT system (Applied Biosystems, Waltham, MA, USA). All primers were synthesized by Tsingke Biotechnology (Beijing, China) and are detailed in Supplementary table 7 of Supplementary Materials.

scRNA-seq

In our study, single-cell RNA sequencing (scRNA-seq) was conducted utilizing orthotopic liver cancer samples of C57BL/6 mice constructed with sgNC and sgTACC3 Hepa1-6 cells. The sequencing was carried out using the 10x Genomics platform (Chromium Single Cell 3’ v3.1, 10x Genomics, CA, USA). First, the cancer samples were dissociated into a single-cell suspension, and the quality inspection and counting were performed to ensure that the cell survival rate exceeded 80%. Library construction was processed using the 10x Genomics Single Cell 3’ Library Preparation Kit (10x Genomics, CA, USA), followed by on-machine sequencing using the NovaSeq 6000 system (Illumina, San Diego, CA, USA). Then, we used the 10x Genomics official analysis software, Cell Ranger (version 3.1, 10x Genomics, CA, USA), to filter, align, quantify, and identify cells from the raw data, ultimately obtaining the gene expression matrix for each cell. Subsequently, the Scanpy tool (version 1.8.2, https://scanpy.readthedocs.io/en/stable/) was employed for further cell filtering, normalization, cell subpopulation classification, differential gene expression analysis, and marker gene calculating. Specifically, we screened high-quality cells based on the following parameters: mitochondrial gene ratio (%) ≤ 10, the number of UMIs (Unique Molecular Identifiers) identified in each cell ≥ 500, and the number of genes identified within each cell ranging among 500-6000. The filtered expression matrix was subjected to dimensionality reduction clustering analysis using Scanpy. The main steps included: data normalization using the LogNormalize method with a target sum of 10,000; selection of 2000 highly variable genes (HVGs) using the dispersion analysis method; and correction of batch effects using the BBKNN (Batch Balanced K Nearest Neighbours) algorithm with default parameters. The data was reduced in dimensionality using principal component analysis (PCA), and then the reduced-dimensional data was passed to UMAP for visualization. After cluster analysis, a total of 17 cell clusters were identified, and these clusters were preliminarily annotated. The identified cell types included T cells, myeloid cells, neutrophils, endothelial cells, hepatocytes, B cells, and fibroblasts. For the identification of tumor cells, inferCNV (version 0.14.1, https://github.com/tallulah/inferCNV) was used to perform copy number variation (CNV) analysis in single-cell transcriptomes. By comparing the CNV gene expression differences between different samples or cell types, the CNV of tumor cells was primarily analyzed. Subsequent analysis, including cell type annotation and trajectory analysis, was commissioned to Hangzhou Cosmos Wisdom Biotechnology Co., Ltd. (Hangzhou, China).

RNA immunoprecipitation (RIP) assay

PLC/PRF/5 and Huh7 cell lines were used for the RIP assay using the EZ-Magna RIP Kit (#17-700, Millipore, USA). In brief, cells were washed using pre-chilled PBS, harvested, and then lysed utilizing a suitable RIP lysis buffer (#17-700, Millipore), supplemented with RNase inhibitors (EO0381, Thermo Fisher Scientific, MA, USA) and a protease inhibitor cocktail (#11697498001, Roche, Switzerland). After incubating on ice for 5-15 minutes, the samples were centrifuged at 12,000 g for a quarter-hour at 4°C, and the supernatant was reserved as the total lysate. Retained 10% of the lysate supernatant as the input control for subsequent RNA quantification and comparison of experimental groups. The remaining supernatant was diluted into NT2 buffer. Next, specific antibodies (anti-LARP1 and anti-PABPC1) were introduced and reacted at 4°C for 4 hours. The immune complexes were obtained using magnetic particles bound to the antibodies. Then, the complexes were digested with proteinase K (20 μg/ml, #E00491, Thermo Fisher Scientific, USA) at 50°C for 30 minutes to release RNA, followed by washing and purification steps using the EZ-Magna RIP Kit. Finally, the enrichment level of ACSL4 mRNA was evaluated using RT‒qPCR with SYBR Green (#11202ES08, YEASEN, China), with IgG antibodies from Abcam (#ab172730, Cambridge, UK) used as negative controls. The information of antibodies used in the RIP assay is listed in Supplementary table 8 of Supplementary Materials.

RNA pull-down assay

The Pierce Magnetic RNA-Protein Pull-Down Kit (ThermoFisher, MA, USA) was used to investigate the interaction between ACSL4 mRNA and LARP1 proteins according to the manufacturer’s protocol. Wild-type and mutant RNA probes complementary to the ACSL4 mRNA region of interest were designed with flanking T7 promoter sequences. RNA probes were synthesized using T7 RNA Polymerase and biotin-labeled, followed by DNase I digestion (1 U/ug DNA, 37°C, 15 min) to remove template DNA. Purified probes were quantified using a NanoDrop spectrophotometer, validated by 1% agarose gel electrophoresis, and stored at −80°C. Next, PLC/PRF/5 cells were lysed in the lysis buffer supplemented with a protease inhibitor cocktail (Cell Signaling Technology) and RNase inhibitor (40 U/uL, ThermoFisher). Biotinylated RNA probes (100 pmol) were immobilized on pre-washed streptavidin magnetic beads. Probe-bound beads were then incubated with cell lysates (4°C, 2 hours with rotation), followed by three washes with the kit’s wash buffer. Proteins were eluted by boiling the beads in 1× SDS (95°C, 10 min) and analyzed by western blotting using anti-LARP1 antibody.

Immunofluorescence

Seed PLC/PRF/5 and Huh7 cells at an appropriate density in confocal dishes (801001, Nest, Switzerland). The next day, fix the cells at RT with 4% paraformaldehyde for 10-15 minutes. Subsequently, use a permeabilization solution (P0097, Beyotime, China) to permeabilize the cells for 5-10 minutes. Next, incubate the cells at room temperature with a blocking solution (P0260, Beyotime, China) for 30 minutes. Then, dilute primary antibodies using the suitable dilution and incubate the cells at 4°C for 12 hours. Dilute the fluorescently conjugated secondary antibody in the suitable dilution, and then incubate the cells at RT for 1 hour. Use DAPI (P0131, Beyotime, China) to stain the cells for 5-10 minutes to visualize the nuclei. After mounting, pictures were photographed using a fluorescence microscope. Detailed information of antibodies employed for immunofluorescence was recorded in Supplementary table 8 of Supplementary Materials. We pseudo-colored the fluorescent dyes conjugated to the PABPC1 antibody.

RNA stability assay

Human HCC cell lines PLC/PRF/5 and Huh7 were seeded in 12-well plates and incubated for 12 hours at 37°C to ensure that the cells reached the appropriate density. On the second day, the cells were administrated with 5 μg/mL of Actinomycin D (#RASP-101, Selleck, Houston, USA) to inhibit RNA synthesis. We gathered cells at predetermined time points, and total RNA was extracted as described above, which was quantified by RT‒qPCR to detect ACSL4 expression. GraphPad Prism software (version 9, GraphPad Inc., USA) was used to plot the decay curve based on the changes in mRNA levels over time.

Dual-luciferase reporter assay

Until reaching 70-80% confluence, human liver cancer cell lines PLC/PRF/5 and Huh7 were grown in the 24-well plate. Indicated plasmids designed by Sangon Biotech Co., Ltd (Shanghai, China) were transfected using Lipofectamine 3000 (L3000008, Thermo Fisher Scientific, MA, USA). Cells were cultured for 24-48 hours to ensure sufficient expression of the transfected reporter gene. The activities of Fluc and Rluc were then measured using the Dual-Luciferase Reporter Gene Assay Kit (11402ES60, YEASEN, China) according to the manufacturer’s instructions. The relative luciferase activity was the ratio of Fireﬂy luciferase activity to Renilla luciferase activity.

ELISA

ELISA assay was carried out in accordance with the manufacturer’s instructions. Information of corresponding ELISA kits is as follows: DHA (abx258057, abbexa, UK), ALT (ab282882, Abcam, UK), AST (ab263882, Abcam, UK).

Co-immunoprecipitation (co-IP)

HEK-293T, PLC/PRF/5, and Huh7 cells were rinsed with pre-chilled PBS and lysed using NP-40 lysis buffer (P0013F, Beyotime, China), supplemented with protease inhibitor cocktail (#11697498001, Roche, Switzerland). The lysate was maintained on ice for half an hour, centrifuged at 12,000 g for 15 minutes at 4°C, and the supernatant was collected as a total protein sample. Next, 200 μg of total protein was reacted with 50 μL of protein A/G agarose beads (pre-incubated with homologous species IgG at room temperature for 1 hour) at 4°C for 1 hour. The beads were then removed by centrifugation. Afterward, 1-5 μg of specific primary antibodies were introduced into the pre-cleared supernatant and reacted at 4°C with gentle rotation overnight. Following this incubation, 30 μL of pre-cooled protein A/G agarose beads were added and incubated at 4°C for an additional 2 hours. The complexes were gathered by centrifugation at 2,500 g at 4°C for 3 minutes and rinsed three times using lysis buffer. Subsequently, 2× SDS loading buffer (containing 100 mM DTT) was added, and the mixture was boiled at 95°C for 10 minutes. The supernatant was then obtained by centrifugation. The eluted product was separated by SDS-PAGE, and targeted proteins were detected by western blotting and subjected to in-gel digestion and LC-MS/MS analysis. The information of antibodies employed in co-IP was recorded in Supplementary table 8 of Supplementary Materials.

Western blotting

The experiment employs a standard western blotting protocol to detect target proteins. Protein extraction was performed using RIPA lysis buffer (P0013B, Beyotime, China) and protease inhibitor cocktail (#11697498001, Roche, Switzerland), and cells were maintained at 4°C for 15 minutes. Following centrifugation at 12,000 rpm for ten minutes, the supernatant samples were collected as the total protein. Protein concentration was calculated with the Pierce BCA Protein Assay kit (23225, Thermo Fisher Scientific, MA, USA). 25 μg protein was mixed with loading buffer and boiled at 99°C for 5 minutes to denature the protein samples. Proteins were loaded onto 10% SDS-PAGE gels (PG112, EpiZyme, China) and subjected to electrophoresis. Membranes were transferred using PVDF membranes (Millipore, USA) and blocked with blocking buffer (P0252, Beyotime, China) for half an hour at room temperature, following incubation with the corresponding primary antibody for 12 hours at 4°C. After washing, strips were reacted with HRP-conjugated secondary antibodies (abs20002/abs20001, Absin, China) for 1 hour at room temperature. Finally, strips were visualized through the ECL kit (PS6009, ProteinBio, Nanjing, China) and imaged with a digital imaging system. The information of antibodies for western blotting assay was recorded in Supplementary table 8 of Supplementary Materials.

IHC and Multiplex IHC (mIHC)

Bake slides with tissue slices at 60°C for 1 hour. Soak the slides in fresh xylene for 10 minutes to dissolve paraffin; repeat 3 times. Perform gradient ethanol treatment: immerse in 100% ethanol for 5 minutes, 95% ethanol for 5 minutes, and 70% ethanol for 2 minutes. For intracellular indicators, add membrane permeabilization agent (P0097, Beyotime, China) and incubate for 15 minutes. Place the dewaxed slides in an antigen retrieval cup and add the appropriate antigen retrieval solution (P0081/P0084, Beyotime, China). Heat in a microwave on high until boiling, then reduce to low heat for 15 minutes to cool. Add enhanced endogenous peroxidase blocking buffer (P0100B, Beyotime, China) and incubate for 10 minutes. Then, add 5% goat serum (SL038, Solarbio, China) diluted in PBS. Add the diluted primary antibody solution and incubate for 1 hour at room temperature with gentle shaking.

For IHC, first, incubate the slides with the diluted solution containing the primary antibody for 12 hours at 4°C. Next, add the HRP secondary antibody working solution (abs50014, Absin, China) to the slides, and the reaction was carried out at room temperature for ten minutes. Then, add 100 μl of the 1× DAB working solution and incubate for 10 minutes or until sufficient color development is observed. Next, counterstain the slides with hematoxylin for 1-2 minutes, following sufficient washing for 5 minutes. Afterward, dehydrate the slides using different concentrations of ethanol washes (70%, 95%, and 100%) for 2-5 minutes each, and clear the slides in xylene. After mounting the slides with neutral gum and coverslips, the stained tissue slices are then observed and analyzed under a light microscope. For the calculation of the H-score, the intensity of staining was classified into four degrees: no staining (0), weak staining (1), moderate staining (2), and strong staining (3). Additionally, the percentage of positive cells in the stained tissues was scored as follows: 0% (0), 1-24% (1), 25-49% (2), 50-74% (3), and 75-100% (4). Multiplying the two together gives the H-score.

For mIHC, incubate the HRP secondary antibody working solution (abs50014, Absin, China) for 10 minutes after the incubation of the primary antibodies. Then, add 100 μl of the 1× dye working solution (abs50014, Absin, China) and incubate for 10 minutes with shaking. Repeat for staining of all antibodies, washing thoroughly with TBST between steps. Finally, add 1× DAPI working solution (abs50014, Absin, China) and incubate for 5 minutes, then add an anti-fluorescence quenching sealing agent. The stained tissue slices are photographed and analyzed through a fluorescence microscope (SP8, Leica, German). The information of antibodies used in IHC and mIHC was listed in Supplementary table 8 of Supplementary Materials.

HE staining

To remove the paraffin, we bake the slides at 60°C for one hour. Soak the slides in fresh xylene for 10 minutes (or longer if necessary) to ensure thorough dissolution of the paraffin; repeat this step three times. Proceed with gradient ethanol treatment which was the same as IHC and mIHC. Rinse the slides thoroughly for 5 minutes to remove ethanol. Stain the slides using hematoxylin for 3-5 minutes to visualize the nuclei. After staining, rinse with water for 5 minutes, and then differentiate in 1% hydrochloric acid in 70% ethanol for a few seconds. Rinse again using tap water for 5 minutes for the removal of any remaining acid. Stain the slides with eosin for 1-2 minutes to visualize the cytoplasm. Dehydrate the slides through different concentrations of ethanol washes, clear the slides, and capture the images of the slides as described previously.


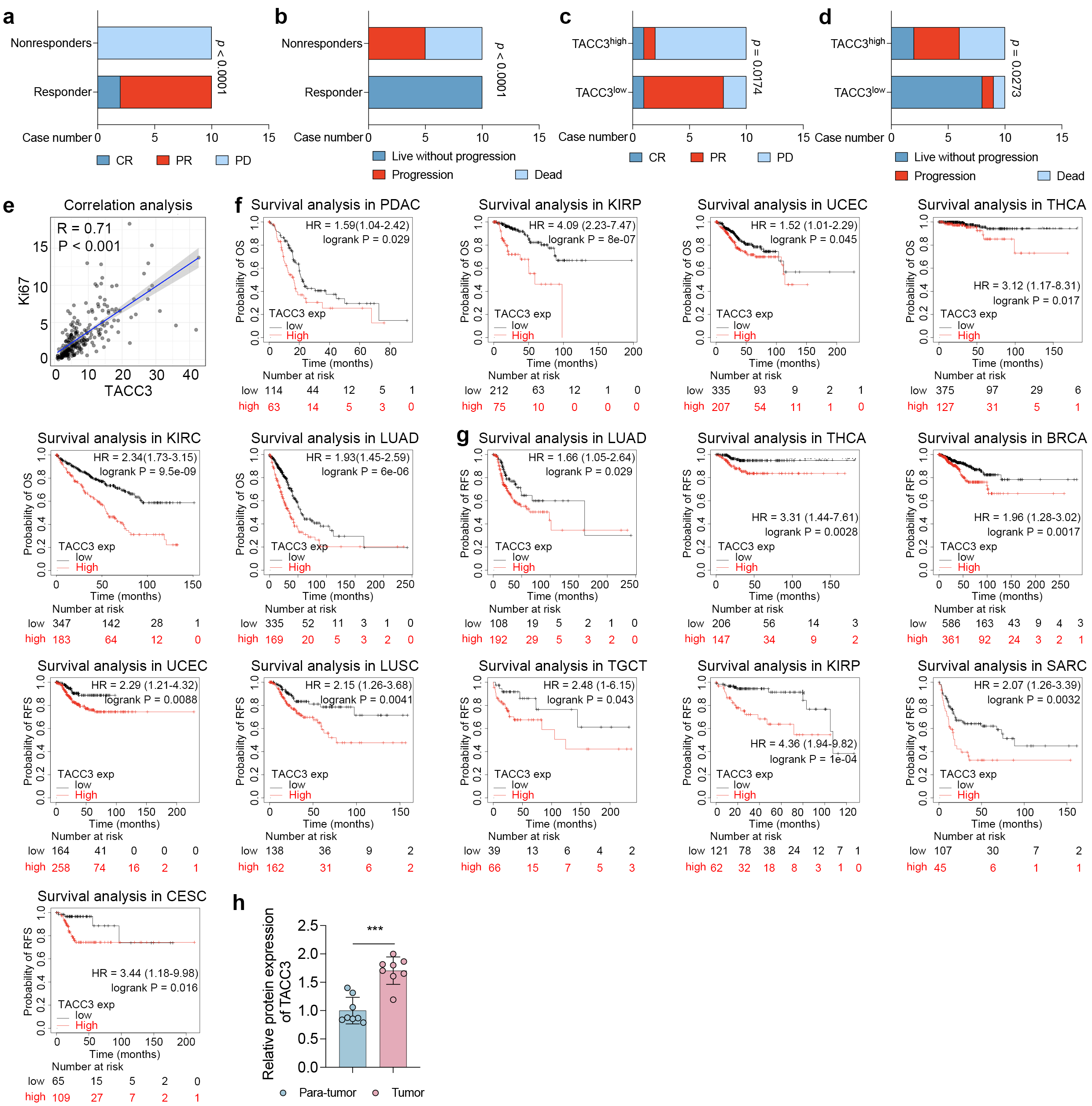


**Figure. S1.** **a‒b** Response criteria (**a**) and survival outcomes (**b**) for 10 immunotherapy responders and 10 non-responders of our center, were evaluated according to RECIST v1.1 guideline. **c‒d** Response criteria (**c**) and survival outcomes (**d**) for 20 patients stratified by the median TACC3 IHC H-score in our immunotherapy cohort. Details of the H-score are provided in the section of Materials and Methods of Supplementary Materials. **e** Correlation of TACC3 expression with Ki67 expression analyzed using ICGC-LIHC dataset. **f‒g** Overall survival (**f**) and relapse-free survival (**g**) curves of TACC3 at the pan-cancer level were obtained from the KM plotter (https://kmplot.com/analysis/). PDAC, pancreatic ductal adenocarcinoma; KIRP, kidney renal papillary cell carcinoma; UCEC, uterine corpus endometrial carcinoma; THCA, thyroid carcinoma; KIRC, kidney renal clear cell carcinoma; LUAD, lung adenocarcinoma; BRCA, breast cancer; LUSC, lung squamous cell carcinoma; TGCT, testicular germ cell tumor; SARC, sarcoma; CESC, cervical squamous cell carcinoma. **h** Quantitative analyses of the western blotting results in Fig. 1h. Data and Error bars were presented as the mean ± SD. Data were analyzed by Chi-square test (a**‒**d), Pearson correlation analysis (e), Log-rank (Mantel-Cox) test (f, g), and Student’s t-test (h). ****p* < 0.001 as compared with the corresponding controls.


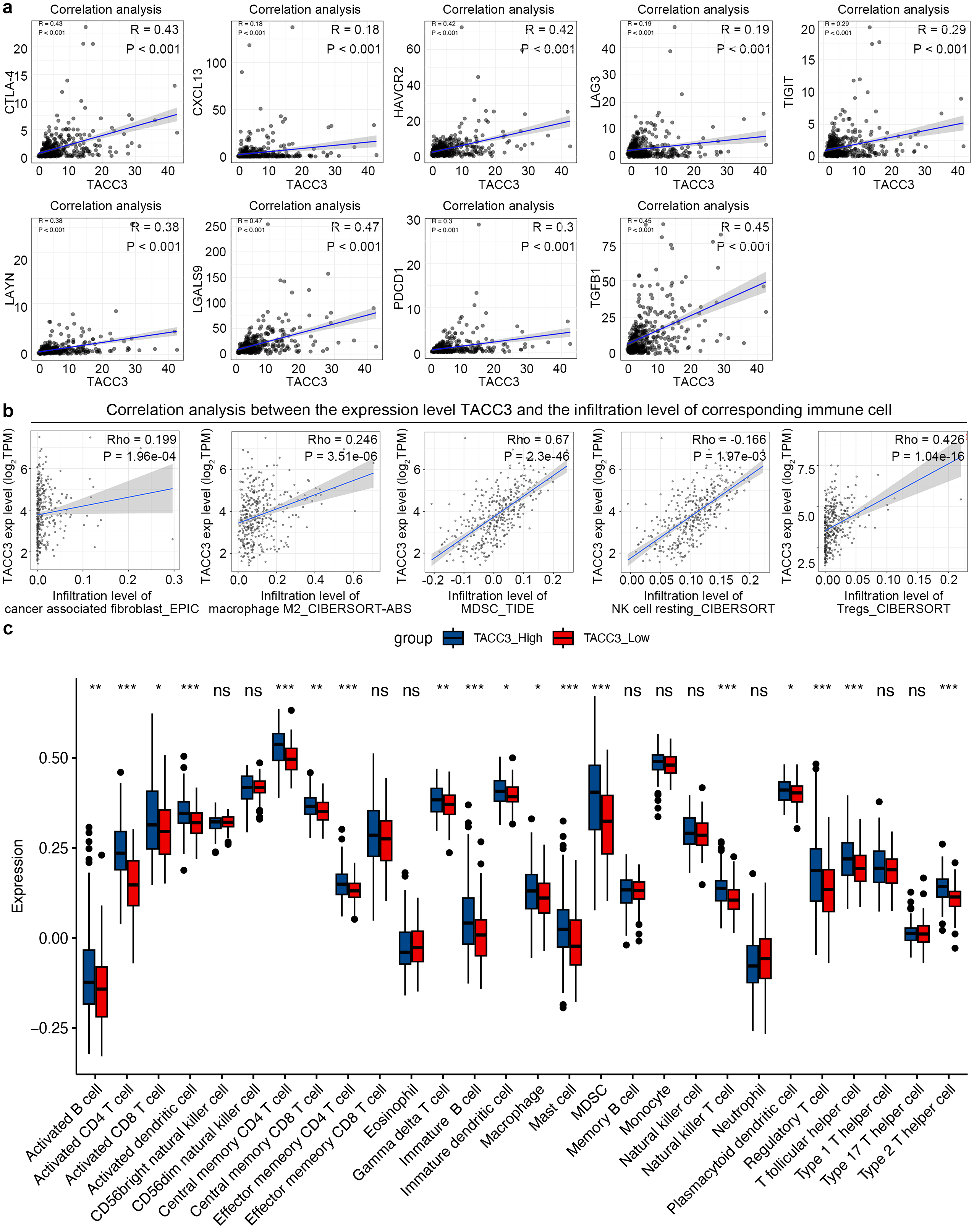


**Figure. S2. a** Correlation of the relative expression of TACC3 to HAVCR2, LAYN, CXCL13, LAG3, CTLA4, LAGLS9, PDCD1, TGFB1, and TIGIT in LIHC analyzed using ICGC-LIHC dataset. Data were analyzed by Pearson correlation analysis. **b** Correlation of TACC3 expression to the infiltration of cancer-associated fibroblasts, M2 macrophage, myeloid-derived suppressor cells (MDSC), and NK cells analyzed by TIMER 2.0 database (http://timer.comp-genomics.org/timer/). The *p* value was calculated by Pearson correlation analysis. **c** ssGSEA analysis was performed using the ICGC-LIHC dataset, and patients were divided into TACC3-high and TACC-low groups according to the median value of the relative expression of TACC3. The markers of immune cells are listed in Supplementary table 9. **p* < 0.05, ***p* < 0.01, ****p* < 0.001 as compared with the corresponding controls.


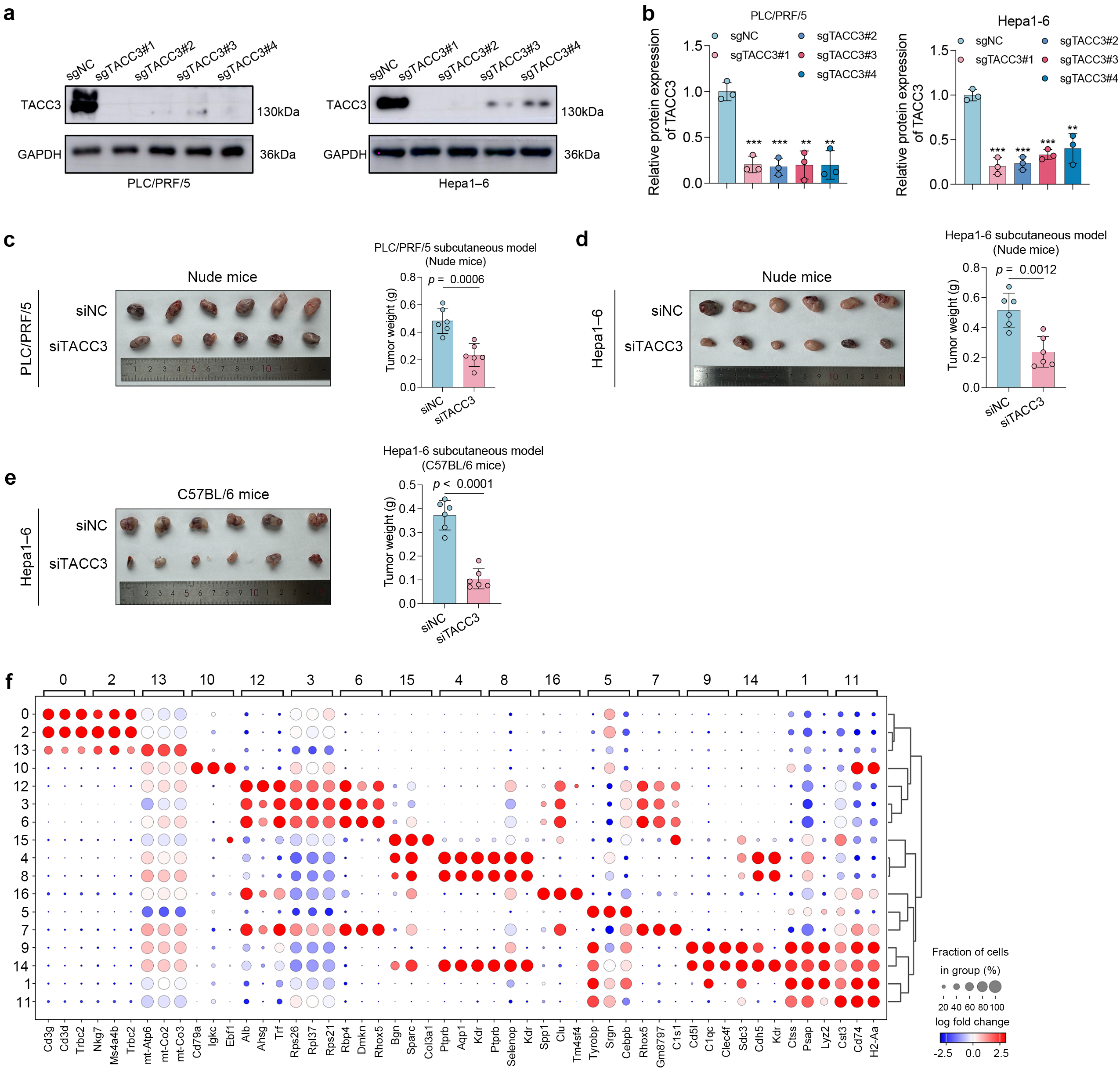


**Figure. S3. a‒b** CRISPR-cas9 technology was utilized to construct the TACC3 knockout cell lines sgTACC3 PLC/PRF/5 cells and Hepa1-6. western blotting was performed to verify the TACC3 knockout effect in the cell pool. Quantitative analyses of the western blotting results (**b**). **c‒e** The representative images of a subcutaneous tumor model, in which PLC/PRF/5 cells (**c**) and Hepa1-6 (**d**) were injected subcutaneously into nude mice and Hepa1-6 were injected subcutaneously into C57BL/6 mice (**e**) to establish the model. When the tumor volume reached 100 mm^3^, the mice were randomly divided into groups and given intratumoral injections of siNC or siTACC3. Statistical analysis was shown in the right panel of the corresponding representative image. n = 6 per group. **f** Cell markers used for annotating cell types in our scRNA-seq analysis. Data and Error bars were presented as the mean ± SD. Data were analyzed by Student’s t-test (b-e). ***p* < 0.01, ****p* < 0.001 as compared with the corresponding controls.


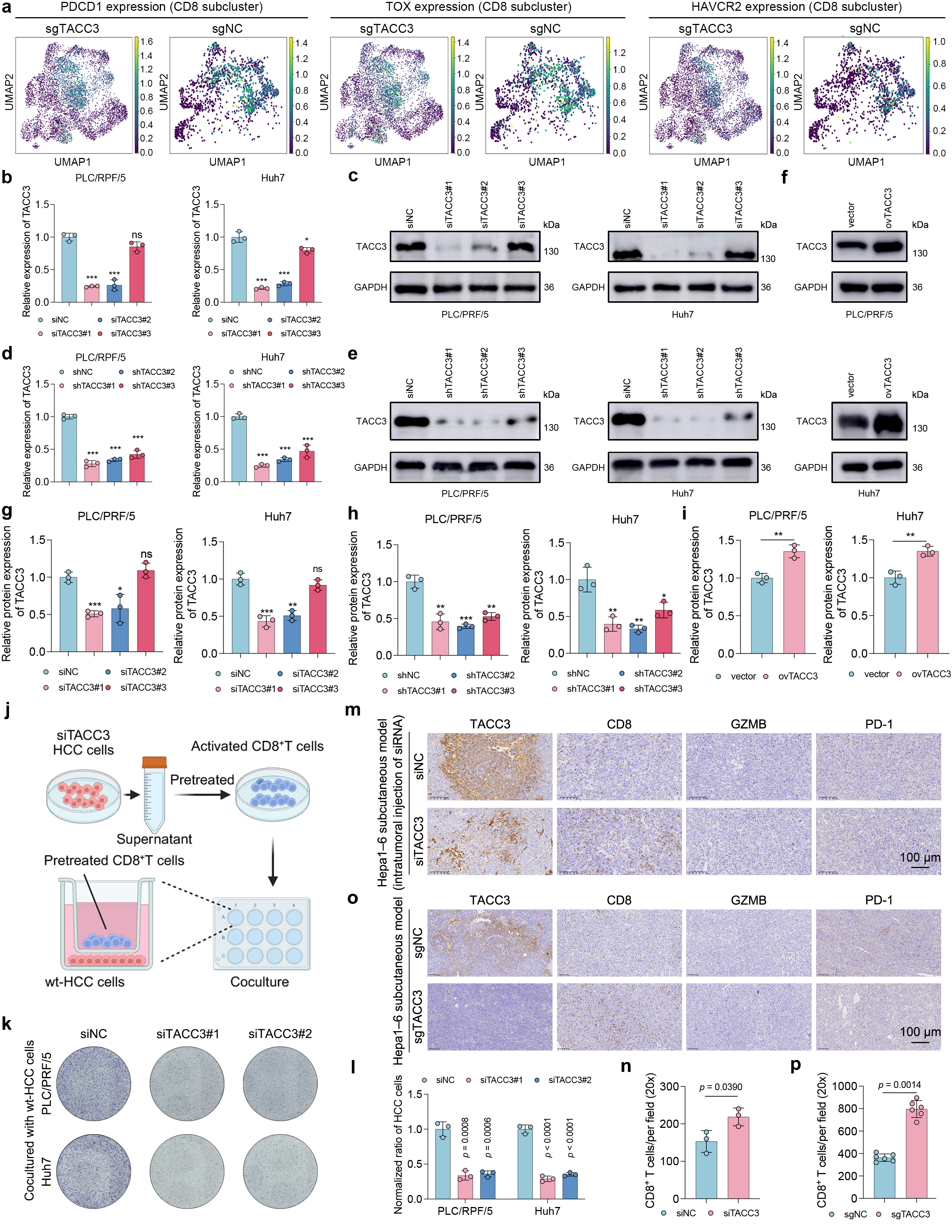


**Figure. S4. a** Feature plots of PDCD1, TOX, and HAVCR2 expression of T/NK cell subsets analyzed using our scRNA-seq data. **b, c** The silencing efficiency of siTACC3 in human HCC cell lines was verified by RT‒qPCR (**b**) and western blotting (**c**). **d‒f** Verification of the construction of stable TACC3-knockdown (**d, e**) and TACC3-overexpression (**f**) human HCC cell lines through RT‒qPCR and western blotting. **g‒i** Quantitative analyses of the western blotting results in Fig. S3c (**g**), Fig. S3e (**h**), and Fig. S3i (**f**). **j** The schematic diagrams of CD8^+^ T cell-mediated tumor killing assay, which was conducted by coculturing activated CD8^+^ T cells and wild-type PLC/PRF/5 or Huh7 cells. Before co-cultivation, activated CD8^+^ T cells were pre-treated with supernatants from PLC/PRF/5 or Huh7 cells transfected with siTACC3. **k, l** Representative images (**k**) and statistical chart (**l**) of CD8^+^ T cell-mediated tumor killing assay for Supplementary Fig. 4j. **m, n** TACC3, CD8, GZMB, and PD-1 IHC analysis of subcutaneous tumor model using wild-type Hepa1-6 cells (n = 3 per group). siNC or siTACC3 were administered by intratumoral injection every 3 days. The statistical analysis of the number of CD8^+^ T cells per 20X field was shown in (**n**). Scale bars, 100 μm. **o, p** TACC3, CD8, GZMB, and PD-1 IHC analysis of subcutaneous tumor model using sgNC/sgTACC3 Hepa1-6 cells (n = 6 per group). The statistical analysis of the number of CD8^+^ T cells per 20X field was shown in (**p**). Scale bars, 100 μm. Data and Error bars were presented as the mean ± SD. Data were analyzed by Student’s t-test (b, d, g-i, l, n, p). **p* < 0.05, ***p* < 0.01, ****p* < 0.001 as compared with the corresponding controls.


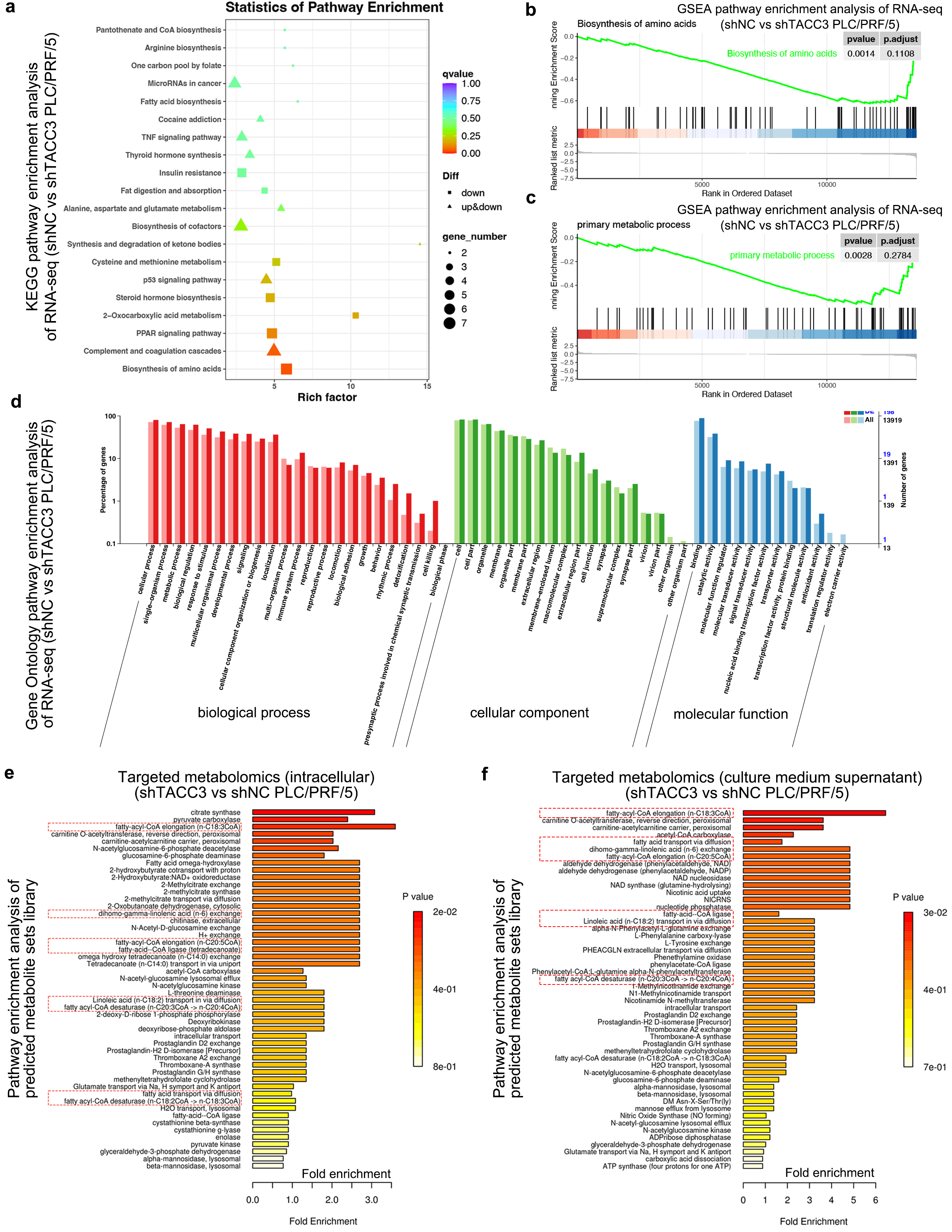


**Figure. S5.** **a‒d** KEGG analysis (**a**) and GSEA analyses (**b‒d**) based on our RNA-seq data using 3 shNC and 3 shTACC3 PLC/PRF/5 cells. **e** Metabolic pathway enrichment analysis based on predicted metabolite-set library using data of intracellular targeted metabolomics. **f** Metabolic pathway enrichment analysis based on predicted metabolite-set library using data of supernatant targeted metabolomics.


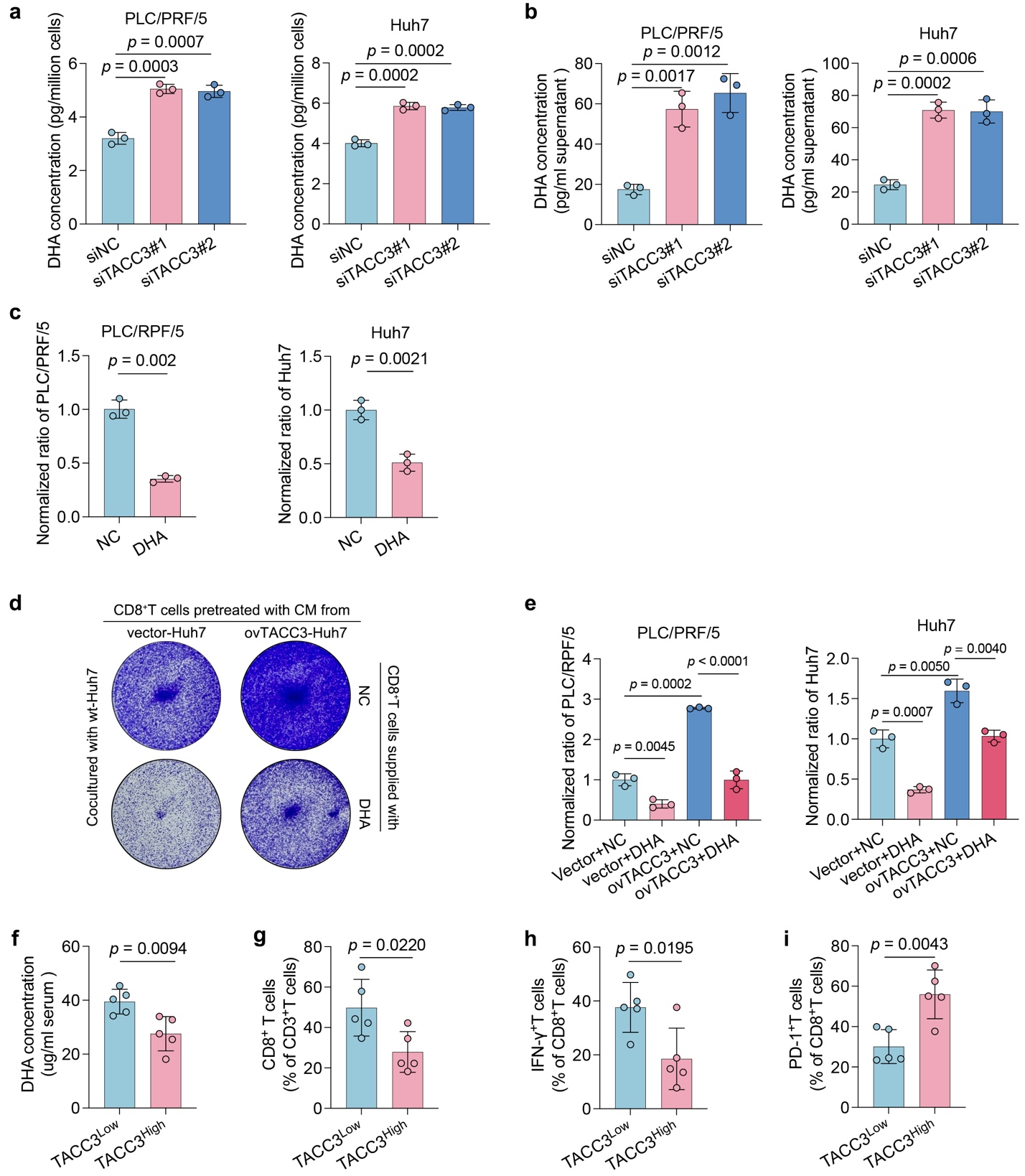


**Figure. S6. a** Intracellular DHA concentration of PLC/PRF/5 and Huh7 cells detected by ELISA. HCC cells were transfected with siTACC3 or siNC. **b** DHA concentration in the supernatant of PLC/PRF/5 and Huh7 cells detected by ELISA. HCC cells were transfected with siTACC3 or siNC. **c** The statistical charts of CD8^+^ T cell-mediated tumor killing assay for Figure 4n. **d** The representative images of CD8^+^ T cell-mediated tumor killing assay, which was conducted by coculturing activated CD8^+^ T cells and wild-type Huh7 cells. Before co-cultivation, activated CD8^+^ T cells were pre-treated with 20um DHA plus the culture medium supernatant from Huh7 cells transfected with vector or ovTACC3. CM, conditioned medium; BSA-ethanol 0.1% v/v was set as negative control (NC) for DHA treatment. **e** The statistical charts of CD8^+^ T cell-mediated tumor killing assay for Figure 4o. **f** Serum DHA concentrations were measured by ELISA in 10 HCC patients grouped into TACC3-high (n=5) and TACC3-low (n=5) cohorts based on average TACC3 mRNA expression of HCC tissues. **g‒i** The total CD8^+^T cell infiltration (**g**), IFN-γ^+^CD8^+^ T cell percentage (**h**), and PD1^+^CD8^+^T cell percentage (**i**) were quantified by flow cytometry in 10 HCC samples from TACC3-high (n=5) and TACC3-low (n=5) patients. Data and Error bars were presented as the mean ± SD. Data were analyzed by Student’s t-test (a, b, c, e**‒**i).


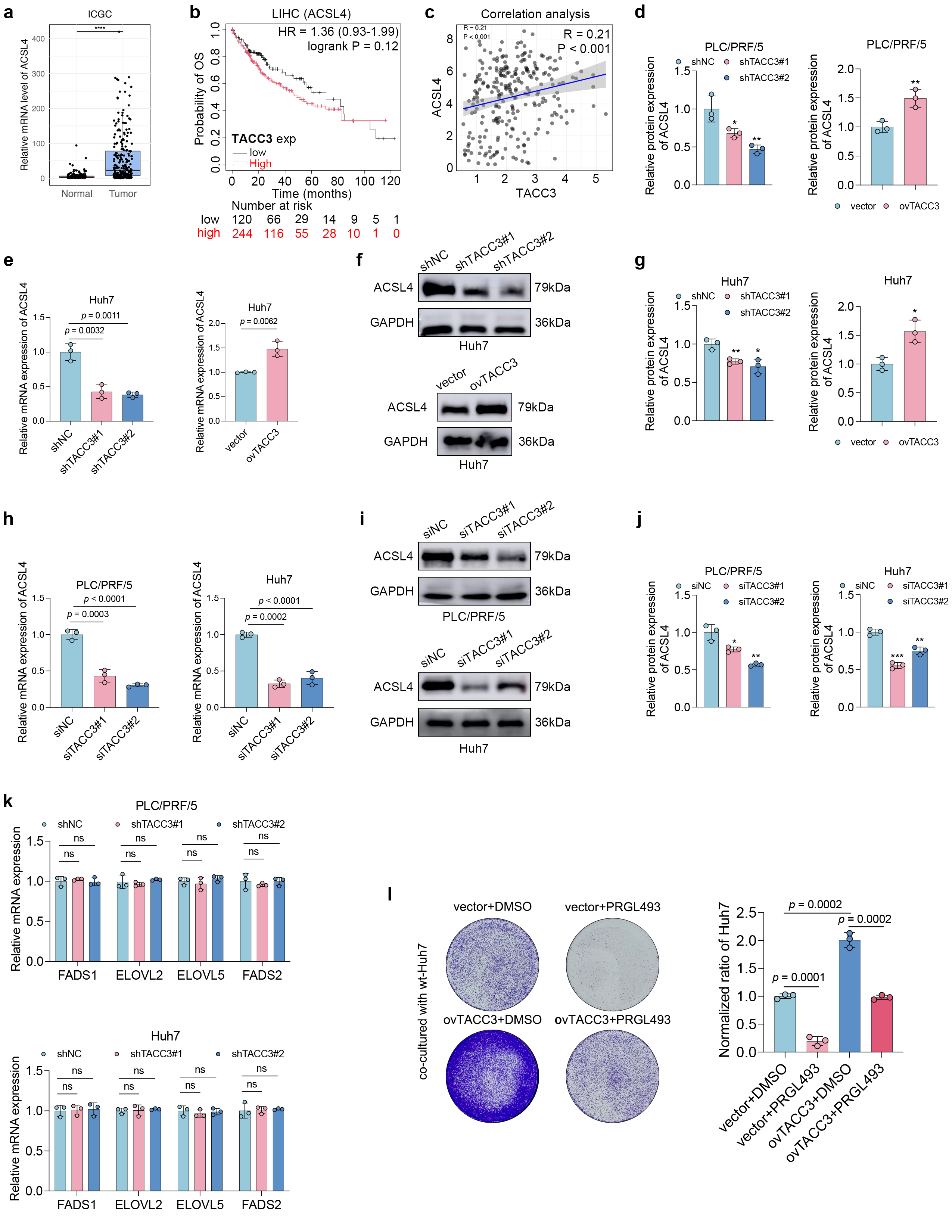


**Figure. S7.** **a** Relative expression of ACSL4 in HCC tumor and normal tissues calculated using ICGC-LIHC dataset. **b** OS curve of ACSL4^high^ and ACSL4^low^ HCC patients drawn by KMplot. KMplot calculated the optimal cutoff value. **c** Pearson’s correlation analysis of the relative expression of TACC3 to ACSL4 by analyzing the ICGC-LIHC dataset. **d** Quantitative analyses of the western blotting results in Figure 5H. **e‒g** Detecting the expression of ACSL4 after stable knockdown or overexpression of TACC3 in Huh7 cells by RT‒qPCR (**e**) and western blotting (**f**). Quantitative analyses of the western blotting results were shown in (**g**). **h‒j** The mRNA and protein levels of ACSL4 in PLC/PRF/5 and Huh7 cells transfected with siTACC3 or siNC were detected by RT**‒**qPCR (**h**) and western blotting (**i**). Quantitative analyses of the western blotting results were shown in (**j**). **k** mRNA expression of multiple enzymes in n-3 PUFA metabolic pathway measured by RT‒qPCR using PLC/PRF/5 and Huh7 cells transfected with shNC or shTACC3. **l** The representative images of CD8^+^ T cell-mediated tumor killing assay, which was performed by coculturing activated CD8^+^ T cells and wild-type Huh7 cells. Before co-cultivation, activated CD8^+^ T cells were pre-treated with the culture medium supernatant from ovTACC3 Huh7 cells treated with PRGL493 (50 μmol). The statistical chart is shown in the right panel. CM, conditioned medium. Data and Error bars were presented as the mean ± SD. Data were analyzed by Student’s t-test (a, d, e, g, h, j**‒**l), Log-rank (Mantel-Cox) test (b), and Pearson’s correlation analysis (c). **p* < 0.05, ***p* < 0.01, ****p* < 0.001 as compared with the corresponding controls.


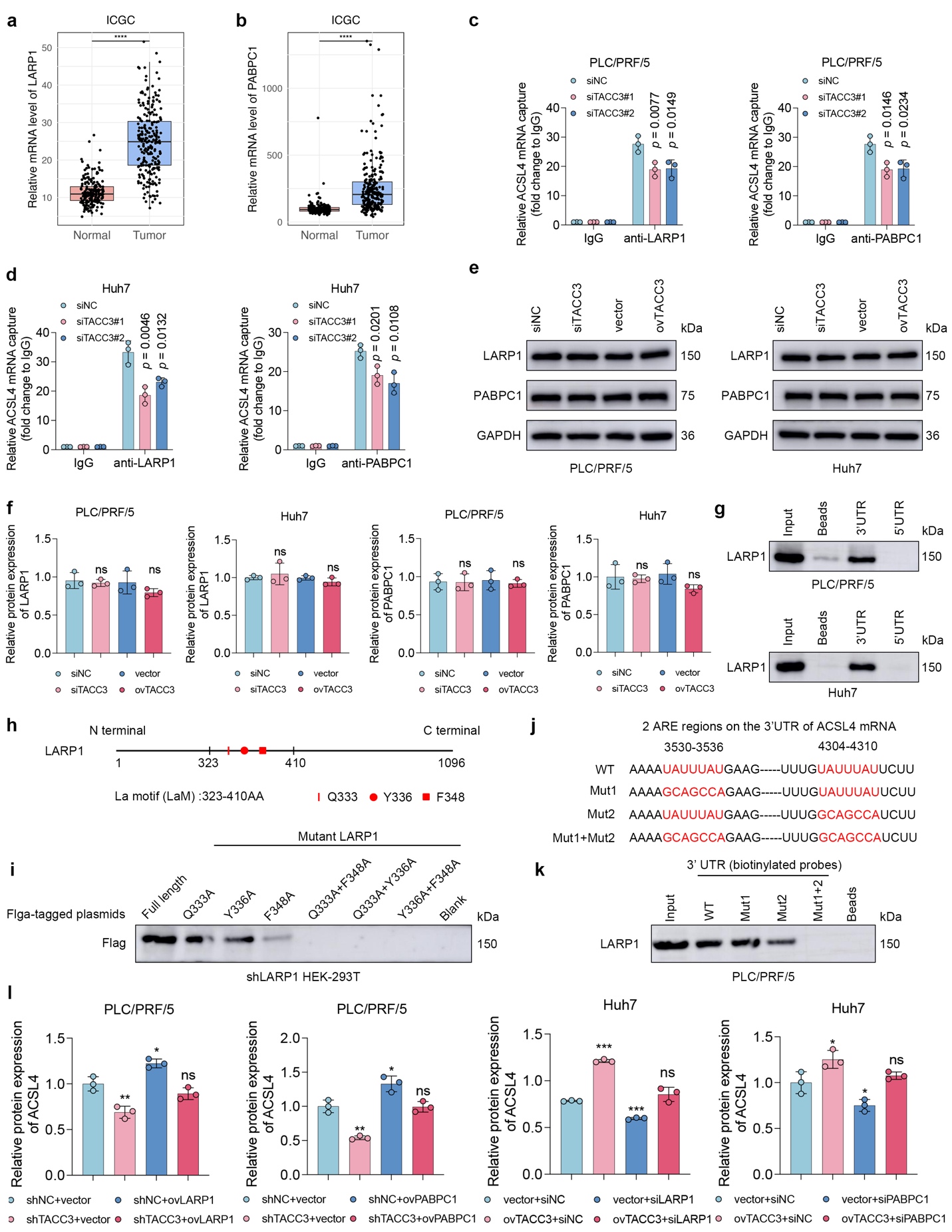


**Figure. S8. a‒b** The relative expression of LARP1 (**a**) and PABPC1 (**b**) in tumor and normal tissues of patients from ICGC-LIHC data. **c, d** RIP assay combined with RT‒qPCR was performed to detect the enrichment of ACSL4 mRNA using PLC/PRF/5 (**c**) and Huh7 (**d**) cells, which were transfected with siTACC3 or siNC. **e, f** LARP1 and PABPC1 protein levels were detected via western blotting (**e**) using PLC/PRF/5 and Huh7 cells transfected with siTACC3 or siNC. Quantitative analyses of the western blotting results were shown in (**f**). **g** RNA-pulldown was conducted to further validate the interaction of LARP1 with the 3'UTR of ACSL4 mRNA. UTR, untranslated region. **h** Schematic diagram of the key amino acids in the La motif on the LARP1 protein responsible for recognizing and binding RNA molecules. AA, amino acid. **i** Plasmids containing wild-type and key amino acid residue mutated LARP1 protein sequences with a Flag tag were constructed and transfected into HEK293T cells with stable LARP1 knockdown. RNA pulldown experiments were then performed using a wild-type ACSL4 3’UTR probe. After elution of the obtained proteins, western blotting experiments were performed using the anti-Flag antibody to verify the identity of the protein. **j** Schematic diagram of wild-type and ARE-mutated ACSL4 mRNA sequences. ARE, AU-rich element. **k** The wild-type and mutant 3’ UTR probes were synthesized by *in vitro* transcription, and then RNA pull-down experiments were performed using lysate from PLC/PRF/5 cell lines. After elution of the obtained proteins, western blotting experiments were performed using anti-LARP1 antibody to verify the binding. **l** Quantitative analyses of the western blotting results in Figure 6m, n. Data and Error bars were presented as the mean ± SD. Data were analyzed by Student’s t-test (c, d, f, i). **p* < 0.05, ***p* < 0.01, ****p* < 0.001 as compared with the corresponding controls.


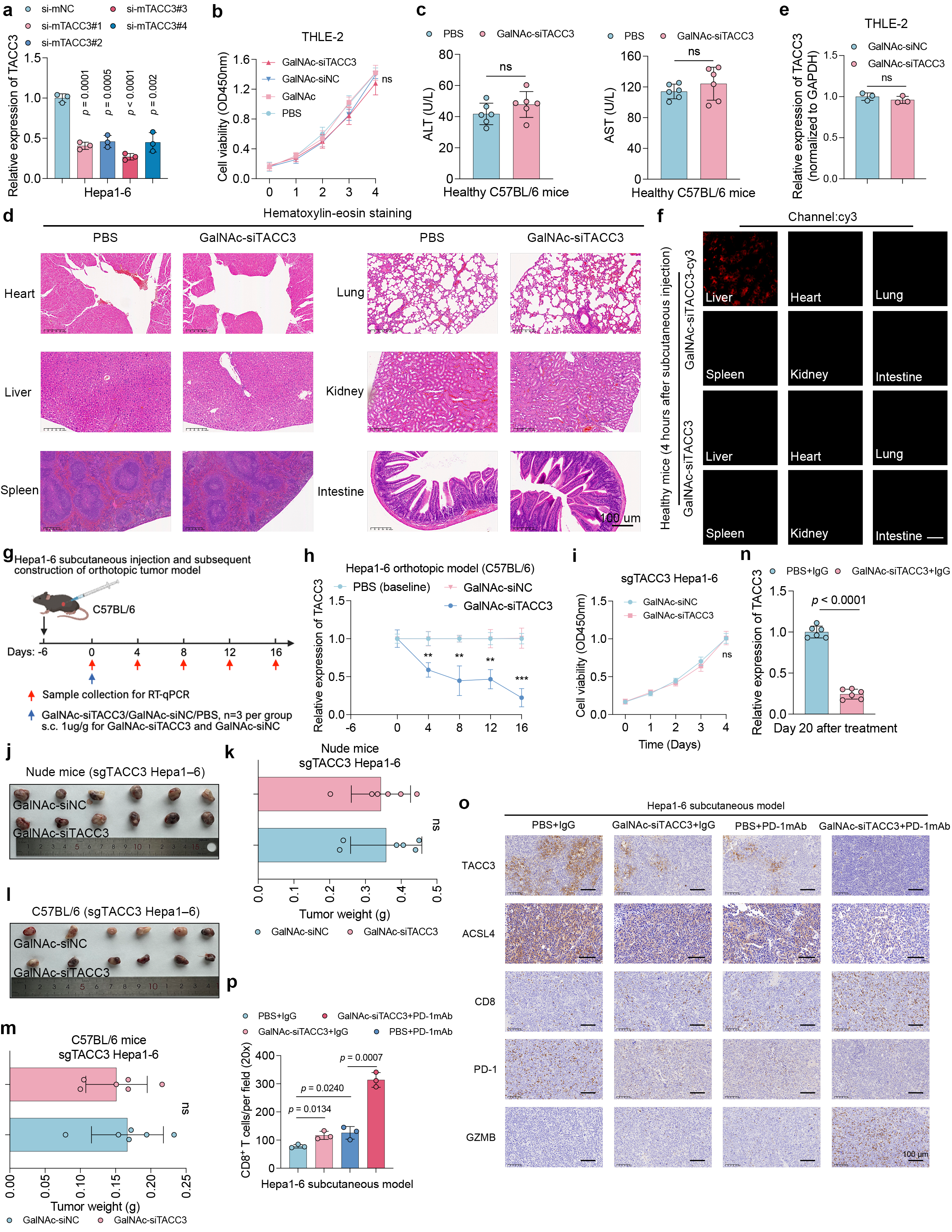


**Figure. S9. a** Verification of the silence efficiency of four si-mTACC3 in Hepa1-6 cells by RT‒qPCR. **b** CCK8 assay was performed in THLE-2 cells treated with PBS, GalNAc, GalNAc-siNC and GalNAc-siTACC3. **c** Serum ALT and AST concentration from healthy C57BL/6 mice treated with GalNAc-siTACC3 quantified by ELISA (n = 6 per group). ALT, alanine aminotransferase; AST, Aspartate Aminotransferase. **d** Representative images of hematoxylin-eosin staining of heart, liver, spleen, lung, kidney, and intestine from healthy C57BL/6 mice treated with GalNAc-siTACC3 or PBS. Scale bars, 100 μm. **e** Relative TACC3 expression was detected by RT‒qPCR using THLE-2 cells treated with GalNAc-siTACC3 or GalNAc-siNC. **f** Healthy C57BL/6 mice were subcutaneously injected with Cy3-labeled or unlabeled GalNAc-siTACC3, and then representative fluorescence images of mouse heart, liver, spleen, lung, kidney, and intestinal sections were observed under a fluorescence microscope. Scale bars, 50 μm. **g** Schematic design of the in vivo silencing dynamics study. **h** Mice bearing orthotopic HCC tumors were treated with a single dose of GalNAc-siTACC3 (1ug/g s.c.), GalNAc-siNC or PBS. Tumor tissues were harvested at indicated time points. TACC3 mRNA levels were quantified by RT‒qPCR. Data are expressed as fold change relative to the PBS group at each time point. n = 3 per group. **i** CCK8 assay was performed in sgTACC3 Hepa1-6 cells treated with GalNAc-siNC or GalNAc-siTACC3. **j‒m** The representative images of the subcutaneous tumor model using nude mice (**j**) and C57BL/6 (**l**) mice were performed in sgTACC3 Hepa1-6 cells treated with GalNAc-siNC or GalNAc-siTACC3. Corresponding statistical charts were shown in (**k**) and (**m**). n = 6 per group. **n** TACC3 mRNA levels were measured by RT‒qPCR using orthotopic tumor tissues from C57BL/6 mice, the treatment of which was described in Figure 6F. **o‒p** Representative images of TACC3, ACSL4, CD8, PD-1, and GZMB IHC analyses of Hepa1-6 subcutaneous model (**o**) and the corresponding statistical analysis of CD8^+^ T cell number (**p**). Scale bars, 100 μm. Data and Error bars were presented as the mean ± SD. Data were analyzed by Student’s t-test (a**‒**c, e, g). **p* < 0.05 as compared with the corresponding controls.


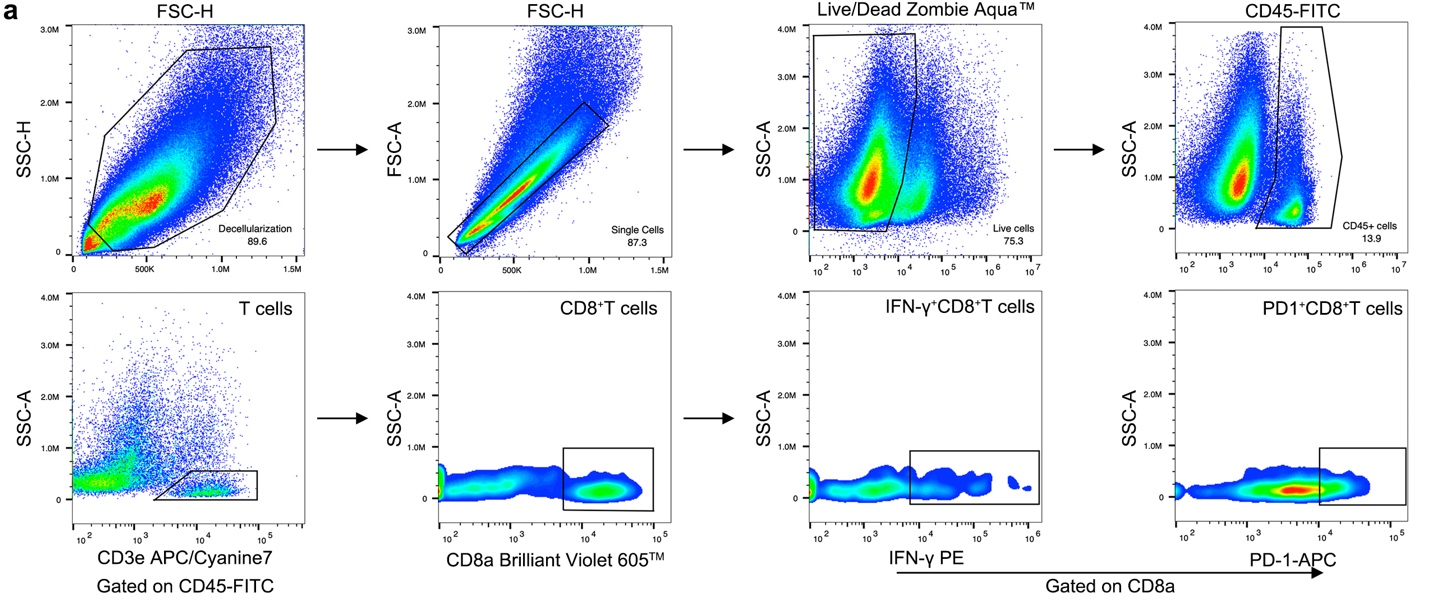


**Figure. S10. a** Flow cytometry gating strategy used in this study that was used to sort total CD8^+^ T cells, IFN-γ^+^CD8^+^ T cells, and PD1^+^CD8^+^T cells.
